# Evolutionary responses to a changing climate: Implications for reindeer population viability

**DOI:** 10.1002/ece3.3119

**Published:** 2017-06-20

**Authors:** Bård‐Jørgen Bårdsen

**Affiliations:** ^1^ Norwegian Institute for Nature Research (NINA) Arctic Ecology Department Fram Centre Tromsø Norway

**Keywords:** agent‐/individual‐based model (ABM/IBM), Arctic/SubArctic, convergence, density dependent selection, dynamic optimization, environmental unpredictability, pastoral livelihood, population dynamics, ungulates

## Abstract

If we want to understand how climate change affects long‐lived organisms, we must know how individuals allocate resources between current reproduction and survival. This trade‐off is affected by expected environmental conditions, but the extent to which density independent (DI) and density dependent (DD) processes interact in shaping individual life histories is less clear. Female reindeer (or caribou: *Rangifer tarandus*) are a monotocous large herbivore with a circumpolar distribution. Individuals that experience unpredictable and potentially harsh winters typically adopt risk averse strategies where they allocate more resources to building own body reserves during summer and less to reproduction. Such a strategy implies that the females do not reproduce or that they produce fewer or smaller offspring. A risk averse strategy thus results in females with large autumn body reserves, which is known to increase their survival probabilities if the coming winter is harsh. In contrast, females experiencing predictable winters may adopt a more risk prone strategy in which they allocate more resources to reproduction as they do not need as many resources to buffer potentially adverse winter conditions. This study uses a seasonal state‐dependent model showing that DD and DI processes interact to affect the evolution of reproductive strategies and population dynamics for reindeer. The model was run across a wide range of different winter climatic scenarios: One set of simulations where the average and variability of the environment was manipulated and one set where the frequency of good and poor winters increased. Both reproductive allocation and population dynamics of reindeer were affected by a combination of DI and DD processes even though they were confounded (harsh climates resulted in lowered density). Individual strategies responded, in line with a risk sensitive reproductive allocation, to climatic conditions and in a similar fashion across the two climatic manipulations.

## 1 INTRODUCTION

Life history theory predicts how organisms make strategic decisions throughout their lifetime (e.g., McNamara & Houston, 1996). A central issue in studies of life histories is to understand how individuals strategically allocate resources between current reproduction and future survival, a trade‐off often referred to as the cost of reproduction (e.g., Williams, 1966). Nevertheless, the combined effect of environmental unpredictability and population density on the cost of reproduction and individual optimization of reproductive strategies is poorly understood. It is well‐known that long‐lived and/or large‐sized organisms tend to favor survival over reproduction, and this has resulted in typically high and stable survival relative to reproduction (Gaillard, Festa‐Bianchet, Yoccoz, Loison, & Toïgo, 2000).

Late winters are bottlenecks for survival for both reindeer (or caribou; *Rangifer tarandus* L.) and other northern large herbivores (e.g., Coulson et al., 2001; Tveraa, Fauchald, Henaug, & Yoccoz, 2003), whereas summer is a period of food abundance (Bårdsen, Næss, Tveraa, Langeland, & Fauchald, 2014; Bårdsen, Tveraa, Fauchald, & Langeland, 2010). In these seasonal habitats, autumn body reserves act as insurance against winter starvation while, in the spring and summer, the females need to make strategic choices on how to allocate resources between increasing their own body reserves and reproduction (e.g., Bårdsen, 2009; Bårdsen et al., 2008). The females may prioritize reproduction, but because reproduction is costly, lactating females are unable to gain as many body reserves during summer as barren ones (e.g., Fauchald, Tveraa, Henaug, & Yoccoz, 2004; Simard, Huot, De Bellefeuille, & Cote, 2014). Additionally, if too many resources are allocated to reproduction, this could jeopardize the female's survival (e.g., Bårdsen et al., 2010)—even though this cost depends on the severity of the coming winter (Bårdsen, Fauchald, Tveraa, Langeland, & Nieminen, 2009). The mechanism behind such a delayed cost of reproduction is that a combination of harsh winters and low autumn body reserves can have negative consequences on both future reproduction and adult survival (e.g., Clutton‐Brock et al., 1996; Tveraa et al., 2003), whereas benign winters may not have beneficial effects of equal magnitude (Bårdsen, 2009). This asymmetry between improved and worsened conditions represents a problem of risk because individuals cannot manipulate the probability of encountering a harsh winter, but may buffer such adverse consequences by reducing their reproductive allocation (for a description of risk sensitive life histories: see e.g., Bårdsen et al., 2008, 2014; Monteith et al., 2013; Morano, Stewart, Sedinger, Nicolai, & Vavra, 2012; Morin, Rughetti, Rioux‐Paquette, & Festa‐Bianchet, 2016).

Rising temperatures and changing precipitation patterns have led to population declines of *Rangifer* (see Pape & Löffler, 2012; Vors & Boyce, 2009) where rain‐on‐snow and freeze‐thaw events, expected to become more frequent with global warming, have negative impacts on reindeer demography and population growth (Hansen, Aanes, Herfindal, Kohler, & Sæther, 2011; Solberg et al., 2001). Yet, not all the predicted effects of future climate change are negative (Pape & Löffler, 2012), which has led to the prediction that the impacts of future climate change on reindeer populations will vary geographically (see Appendix S1 and references therein). Of special importance is that when faced with a higher degree of environmental unpredictability, individuals may adopt more risk sensitive strategies, which again affects demographics such as survival and reproduction. Consequently, risk sensitivity provides us with a theoretical framework for understanding how a changing climate may influence the population dynamics of long‐lived mammals.

Studies of evolutionary changes are conducted at various timescales, evolutionary responses might be genotypic or phenotypic, and evolution is relevant for understanding virtually all aspects of biology—such as understanding how climate change affects both single species and biodiversity in a broad sense (Hoffmann & Sgro, 2011). It is, however, challenging to assess the fitness of different strategies in environments where both density dependent and density independent factors are acting (e.g., Heino, JaJ, & Kaitala, 1998). In empirical studies, this challenge arises due to the need for longitudinal data, preferably from a wide range of different environments, lasting long enough to assess evolutionary responses (e.g., Clutton‐Brock & Sheldon, 2010). Based on data from reindeer populations experiencing contrasting environments, it has, for example, been shown that the effects of density and climate must be interpreted together; harsh winters might have larger negative effects on female reproductive allocation at high density compared with low density (Bårdsen et al., 2010, 2014). Moreover, the development of pure analytical models assessing the simultaneous effects of climate and density is also challenging (McNamara, 1997; McNamara, Webb, & Collins, 1995) due to a process known as density‐dependent selection, which means that a feedback between genotypes and density occurs (Travis, Leips, & Rodd, 2013). Such density‐dependent selection might, for example, occur if low‐density environments support genotypes giving rise to risk‐prone strategies where individuals are smaller and allocate more of their resources to reproduction (and less to survival) compared to high‐density environments resulting in more risk‐averse strategies. Computer simulations can, at least to some extent, overcome these challenges as they enable investigations over time‐scales sufficiently long to allow evolution to act at the same time as a wide range of different life history strategies can play against each other. In this respect, individual‐ or agent‐based models (IBMs/ABMs: e.g., Grimm et al., 2010) can be useful. Their utility is based on a set of autonomous individuals capable of interacting with each other as well as with their environment according to certain behavioral rules (e.g., Billari, Fent, Prskawetz, & Scheffran, 2006). In contrast to more traditional forms of modeling, ABMs offer the possibility to model individual heterogeneity (e.g., Gilbert, 2008); for example, at a given point in space and time, an individual's trait, such as how many resources it allocates to reproduction, may be defined by a particular genotype affecting a reaction norm for allocation. In this respect, ABMs are designed to mimic natural biological populations where individuals—among other things—are born, reproduce (or not), and eventually die. Large‐scale system patterns, that is, macro‐level patterns like population abundance, then arise from individual traits, interactions between individuals, and environmental conditions (so‐called emergent properties: see, e.g., Deangelis & Grimm, 2014).

Consequently, ABMs have equipped scientific disciplines, such as ecology, epidemiology, and social sciences, with a powerful tool for answering complex questions in an understandable way. ABMs have, for example, increased our understanding of basic biological processes (review: Deangelis & Grimm, 2014) including forest dynamics (review: Bugmann, 2001); spatial dynamics of terrestrial mammals (e.g., Marucco & Mcintire, 2010); bird breeding synchrony (e.g., Jovani & Grimm, 2008); and the spread of diseases in host populations (e.g., Eisinger & Thulke, 2008). Of particular relevance to this study is the ability for ABMs to realistically model the evolution of life history strategies in environments where both density‐ and density‐independent processes affect individuals with different states (e.g., Mysterud & Bischof, 2010).

**Figure 1:**
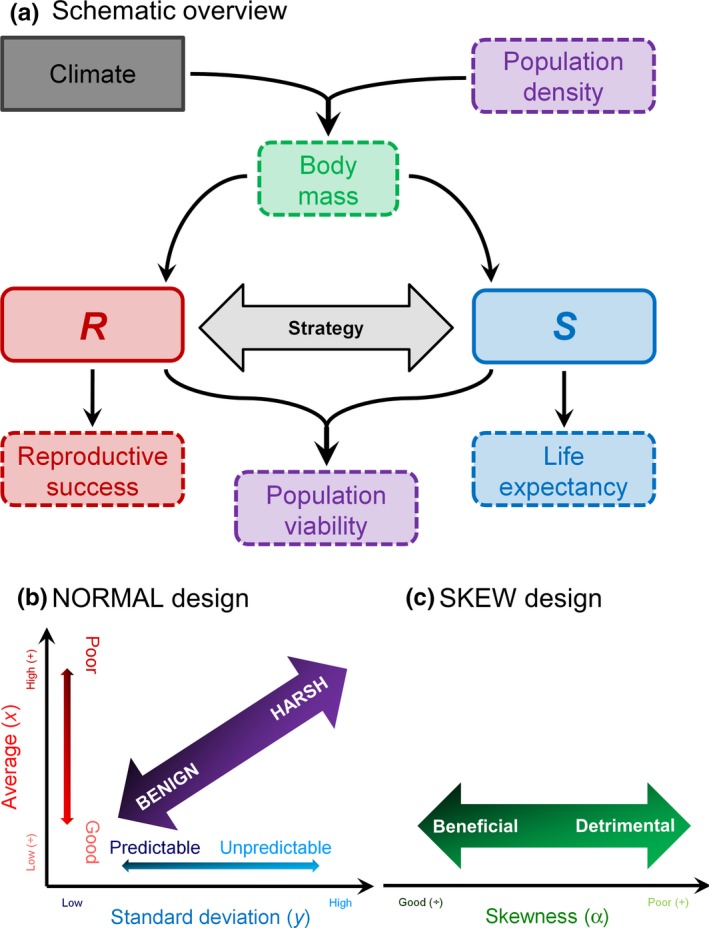
Schematic diagram of the model (a) showing the link between the reproductive strategies and population dynamics. Boxes with dotted lines indicate parameters commonly estimated in empirical studies, whereas solid line boxes denote where the strategy adopted by the different individuals differs: *R* and *S* represent theoretical properties denoting the amount of resources allocated to reproduction and to own body reserves (a survival proxy), respectively. The thick arrow indicates the cost of reproduction: that is, the trade‐off between *R* and *S* as *S* = 1 − *R*. Conceptual illustration of the design of the “computer experiment” where manipulations were performed through gradients in climatic conditions for normally distributed (NORMAL, b) and skew‐normally distributed (SKEW, c) climatic conditions (Appendix S1)

This study is a follow‐up to the ABM by Bårdsen, Henden, Fauchald, Tveraa, and Stien (2011) where the female segment of reindeer populations inhabiting contrasting environments was examined (Figure 1a). In both models, body mass is a state variable, but its importance varies seasonally (see also, e.g., Bårdsen et al., 2014). The present model, however, develops and improves Bårdsen et al.'s (2011) implementation of reproductive strategies and uses more realistic scenarios for future climate change. The overall aim of this study was thus to test the extent to which the evolution of life histories and population dynamics is affected by climatic conditions. Specifically, I assess how climate affects (1) the evolution of both genotypic traits and phenotypes affecting reproductive allocation strategy. I also want assess (2) if greater variability in genotypic traits, that is, a relaxation of one of the key assumptions in Bårdsen et al.'s (2011) model, results in a more plastic reproductive allocation. Furthermore, I assess (3) whether such plasticity results in individuals with better climatic buffering capacities; and (4) populations that are less vulnerable to poor winters. Finally, I assess (5) if a more up‐to‐date scenario for future climate change, where the frequency of good and poor years increases (Appendix S1), imposes any effects on the outcome from the model.

## 2 METHODS

### 2.1 Model description

Time (*t*) was discrete, one time step equaled one year, and the model was run *T* time steps, from *t* = *t*_0_ to *t* = *t*_0_ + *T*. During summer, which is the season when females chose how many resources to allocate to reproduction, they do not know what the coming winter's environmental conditions will be. This means that even though some processes affecting individuals in one season will cause lagged effects, these processes happen independently of each other. The rationale for this was discussed in earlier studies assessing risk‐sensitive life histories (e.g., Bårdsen et al., 2008, 2009, 2010). Consequently, each time step was divided into two seasons: (1) summer, where individuals accumulate body mass, reproduce and experience density‐dependent body mass development; and (2) winter, where individuals experience stochastic climatic conditions that affect their body mass development and survival. Individual state variables included age (*j*; year), body mass (kg), and the three genotypic traits defining the reproductive allocation strategy, whereas population‐level state variables included summer density (*D*; individuals km^−2^; an emergent property) and winter climatic conditions (*E*). A model description following the “overview, design concepts, and details” protocol commonly applied in ABMs (Grimm et al., 2006) is in Bårdsen et al. (2011: Appendix 1). The present model differs from the model in Bårdsen et al. (2011) in how strategies were defined, how convergence was assessed, and how some of the output was analyzed.

### 2.2 Reproductive strategies—genotypic traits versus phenotypes

**Figure 2:**
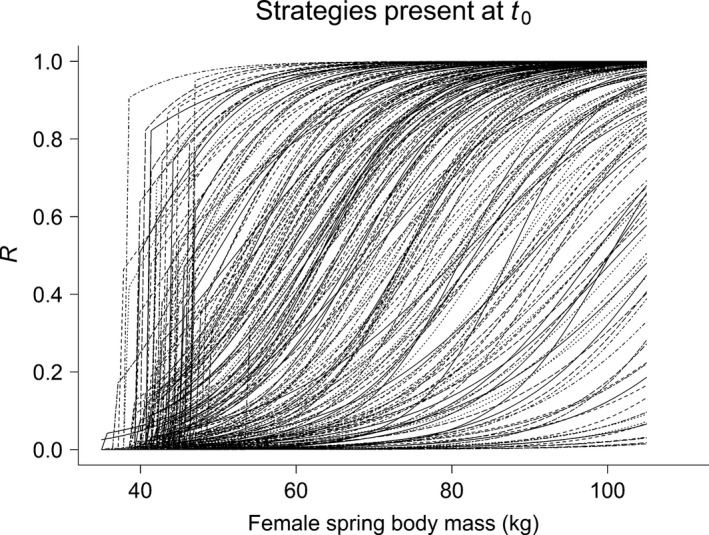
Between‐individual variability in strategies present in the population when the simulation was initiated (i.e., at *t *= *t*_0_) showing reproductive allocation (*R*) as a function of female spring body mass (see also Appendix S2). The three different genotypic traits define individual strategies (each line represents one female) as follows: the intercept (*a*_*R*_), slope (*b*_*R*_), and the threshold body mass (γ_*R*_)

In the model, increased reproductive allocation (*R*) induces a cost to survival (*S*, where *S* = 1 − *R*) and a benefit to offspring survival. For a constant spring body mass, this cost increases at high density and during harsh winters (see fig. 2 in Bårdsen et al., 2011). The relationship between *R*, which is the strategies phenotypic expression, and female spring body mass (Spring_bm_) had a logistic form. This reaction norm was defined by three genotypic traits (i.e., parameter values defining the individual strategies), intercept (*a*_*R*_), slope (*b*_*R*_), and a threshold body mass (γ_*R*_), as follows (Figure 2):

(1) $$R=\frac{1}{1+e^{-\left[a_{R}+b_{R}\times {Spring}_{\mathrm{bm}}\right]}}\;\text{if}j\;\;>1\;\&\;\;\text{if}\;\;{\text{Spring}}_{\mathrm{bm}}>\gamma_{R}$$

(2) $$R=0\;\;\;\;\text{if}\;j\;\;\leq 1\;\;\text{or}\;\;\text{if}\;\;{\text{Spring}}_{\mathrm{bm}}\leq \gamma_{R}$$

This means that neither juveniles (*j* ≤ 1) nor individuals below the threshold spring body mass (γ_*R*_) allocated resources to reproduction. Such a threshold body mass has previously been documented for large herbivores, including reindeer (e.g., Skogland, 1985), and even though this parameter was subject to evolution, its initial value was empirically based (see Appendix S1 for details). The separation of traits into genotypic and genotypic traits should not be interpreted in a literal sense. I simply use these terms to separate between the outcome of a strategy (the phenotypic trait), which may vary in time for the same individual, and the parameters defining a strategy and thus the reaction norm. I refer to these parameters as genotypic traits as they are constant in time for the same individual (eqs. (1), (2)). Within this framework, the phenotypic trait is a result of the interaction between the genotypic traits and spring body mass that may be viewed as an environmental proxy (even though it is also affected by other factors). If a female allocated resources into reproduction, spring body mass was an important state variable, which was justified because body mass is a positive predictor for both survival and reproductive output for female reindeer. Additionally, senescence was not implicitly included in the model, but an upper limit of 16 years of age was included to ensure that no females became unrealistically old. In the previous model, the only source of within‐strategy variability was *R*, which could vary between years even for the same individuals, whereas the only source of between‐strategy variability was the values used for *b*_*R*_ as the other genotypic traits were fixed: *a*_*R*_ = −10.00 and τ_spring_ = 43.20 (Bårdsen et al., 2011: Appendix 1). A key difference between the studies is thus that in the present model all three genotypic traits were simultaneously allowed to evolve. This was implemented as an individual (*i*) was born with a value for a given genotypic trait as follows:

(3) $${\text{Genotype}}_{i}={\text{Genotype}}_{\mathrm{Fem}}+{\text{Genotype}}_{\mathrm{error}}.$$

Consequently, the genotypic traits (Genotype_*i*_ which substitutes *a*_*R*_, *b*_*R*_ and τ_spring_) for a new‐born was inherited from its mother (Genotype_Fem_), but a small variability (Genotype_error_) was added to the value inherited by the offspring. To ensure individual variability in all three traits, the realized value for a given individual was a random number drawn from a normal distribution:

(4) $${\text{Genotype}}_{{\mathrm{error}}_{i}}\approx N\left(0,\;\;{\text{Genotype}}_{\mathrm{SD}}\right).$$

The magnitude of this error ensured that the correlation between mother and offspring was ~1, but ≠1. *A priori*, the standard deviations (Genotype_SD_) for each trait were set to be 500 times smaller than the average values for the most winning strategy in Bårdsen et al. (2011), and were thus equal to 0.0200, 0.0020, and 0.0864 for *a*_*R*_, *b*_*R*_, and τ_spring_,  respectively. The overall rationale for introducing some level of randomness to the inherited values was to avoid transient effects. In the absence of any random noise, some strategies (i.e., certain combination of genotypic traits) going extinct in the initial phase of a simulations could never re‐appear again later on.

### 2.3 Body mass as a state variable

As in the previous model, body mass development occurred from one season to the next with spring and autumn body mass acting as states affecting reproduction and survival, respectively (see p. 8–10 in Bårdsen et al., 2011: Appendix 1 for technical details). First, summer body mass development was modeled in a linear fashion through a gain function and a basal metabolic rate. The gain function represents the per unit female spring body mass increase over summer and was subject to negative density dependence and how density interacted with a given allocation. Consequently, the individual's phenotypic expression, that is, *R* (eq. (1), (2)) and *S*, had positive effects on offspring and female body mass gain, but this positive effect was restricted by population density. At high density, body mass gain would approach 0 even if *R* or *S* was 1 (this happened at 6.5 animals km^−2^, which was based on the range in observed densities for Norwegian reindeer populations), whereas the gain would approach *R* or *S* as density approach 0. Gain, which was defined between 0 and 1, affected autumn body mass positively, whereas autumn body mass was restricted by the following: (1) A summer basal metabolic rate, which was a function of spring body mass; and (2) an upper body mass threshold (provided in Table A1.2 in Bårdsen et al., 2011). Second, the only mortality during summer was that individuals below an unrealistically low autumn body mass threshold (15 kg) were assumed to have died of starvation.

Third, winter survival followed a logistic form and was modeled as a function of autumn body mass, winter climatic condition, and an interaction between them. Large individuals experienced better survival probabilities than small ones, and the chance of survival was lower in poor than in good winters. The interaction, however, ensured that the survival of larger individuals was less sensitive to climate relative to smaller ones (e.g., Tveraa et al., 2003), which means that survival was state depended. Fourth, all surviving animals experienced a proportional loss of their autumn body mass during winter, which also was defined as a logistic function. This proportional loss was affected by climate, but in contrast to summer mass development, it was not subject to density dependence, which means that individuals lost more body mass in poor as opposed to good winters.

### 2.4 Running the model and interpreting results

#### 2.4.1 Initiation, climatic scenarios and convergence

Simulations were initiated by creating 200 individuals with similar age (2 years) and large contrasts in body mass and genotypic traits (Appendix S2, Figure 2). I ran two sets of computer experiments, or scenarios, for winter climatic conditions (*E*; Appendix S1) a: (1) Normally distributed environment (NORMAL) in which *E* was modelled with white noise (Figure 1b) as in Bårdsen et al. (2011); and (2) skew‐normally (Azzalini, 2005) distributed environment (SKEW) where *E* was generated with increased frequency of poor or good environments (Figure 1c) without generating extremes far outside currently observed conditions (Appendix S1: Fig. S1.1). The latter was justified as scenarios for future climate change also predict increased frequencies of extreme events: A trend already observed in empirical time series (see Appendix S1 and references therein). As suggested by Bårdsen et al. (2011), this type of change cannot be simulated by changing the parameters of the normal distribution. Due to the way strategies were allowed to evolve, convergence was achieved when evolution was assumed to have stabilized the distribution of the three genotypic traits defining the reproductive strategies: That is, showing no temporal trends even though the different genotypic traits were allowed to be correlated (Appendix S3).

#### 2.4.2 Interpreting output: pseudo‐empirical statistical analyses

Data on both individual‐ and population‐level characteristics were “collected” 200 years after convergence in each simulation. These data were treated as empirical data and analyzed using generalized additive models (GAMs), which is a standard statistical methodology (see Appendix S3 for technical details). I chose to use GAMs as the degree of complexity, or smoothness, is selected objectively (Wood, 2006), which is an advantage in ABMs where emergence is one of the properties being assessed. In these analyses, the relative importance of climate, that is, the estimated variability [$\hat{\text{sd}(E)}$], average ($\hat{E}$) and skewness ($\hat{\alpha }$) of the generated climatic variable, and density ($\hat{D}$) were assessed with respect to predicting: (1) reproductive strategies (${\hat{R}}^{}$); (2) commonly used empirical measures of individual life histories (e.g., age, body mass, and reproductive success); and (3) population dynamics based on estimates from second‐order autoregressive [AR(2)] models (Appendix S3 provides details about the time series analyses). As in the previous model, the comparison of the coefficients from the AR(2) models with empirical analyses was the main motivation for choosing this approach (similar empirical time series analyses has been performed at a large spatiotemporal scale in both Norway and Sweden: Bårdsen, Næss, Singh, & Åhman, 2017; Tveraa et al., 2007). For all analyses, this resulted in fitting a density‐independent (DI) and a density‐dependent (DD) model to each response separately (Appendix S3).

## 3 RESULTS

### 3.1 Normally distributed environments (NORMAL)

**Figure 3:**
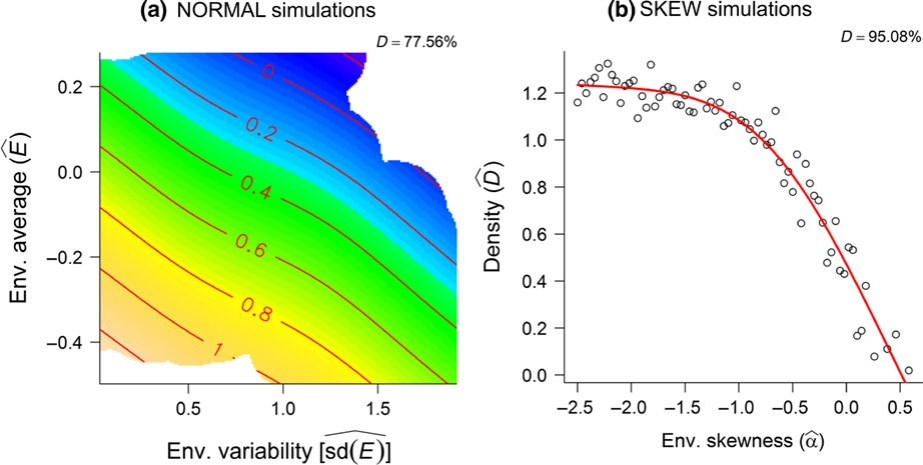
Generalized Additive Models (GAMs) showing how population density ($\hat{D}$) was a function of: (a) the interaction between environmental variability and average for the NORMAL simulation; and (b) environmental skewness ($\hat{\alpha }$) in the SKEW simulation (Appendix S4)

Both the phenotypic expression of the strategies, that is, the amounts allocated to reproduction ($\hat{R}$), and the corresponding genotypic traits, that is, the intercept ($\hat{a_{R}}$), slope ($\hat{b_{R}}$), and body mass threshold ($\hat{\gamma_{R}}$), were affected by both winter climatic conditions and population density (Appendix S4 provide details of the analyses pertained to the NORMAL simulations). The relationship between climatic conditions and $\hat{R}$ was, however, positive and the opposite of that found in the previous study, which was unexpected as climate, was modeled on a relative scale where “less is better” (Appendix S1), whereas its relationship with density was as expected negative and similar to that in Bårdsen et al. (2011: fig. 3). Nonetheless, the DD model explained more of the variance in $\hat{R}$ compared to the DI model. This may indicate that density is a more powerful force affecting reproductive allocation compared to climate (see also Bårdsen, 2009: Paper 4) or simply that density was confounded with climate (density was highest in the most favorable environments: Figure 3a).

Reproductive success ($\hat{rs}$), that is, the number of offspring female^−1^, decreased along the benign‐harsh environmental gradient as $\hat{rs}$ was negatively related to both environmental average ($\hat{E}$) and its standard deviation [$\hat{\text{sd}(E)}$]. Females produced more offspring in high‐ versus low‐density habitats. Offspring spring and autumn body mass increased along the benign‐harsh environmental gradient, but showed evidence of negative density dependence. Reproductive success showed similar trends as in the previous study, whereas the effect of $\hat{E}$ on offspring body mass was positive and the opposite to what Bårdsen et al. (2011: fig. 4) found, whereas $\hat{\text{sd}(E)}$ was a positive predictor of offspring body mass. Moreover, the finding that spring body mass was higher than autumn body mass was similar across studies.

Female age decreased along the benign‐harsh environmental gradient, but animals became older as density increased. This was in contrast to the previous study where female age increased along the benign‐harsh environmental gradient. In the autumn, female body mass increased along the benign‐harsh environmental gradient and showed clear evidence of negative density dependence. In the spring, however, the smallest females were found in poor and predictable environments, which was the opposite of autumn body mass. Additionally, no density dependence was documented in the spring. Adult body mass showed similar trends as in Bårdsen et al. (2011: fig. 5).

The direct negative effect of density on population growth, that is, the effect of direct regulation ($\hat{1-\beta_{1}}$) from the autoregressive models, decreased along the benign‐harsh gradient as $\hat{1-\beta_{1}}$ was positively related to both $\hat{E}$ and $\hat{\text{sd}(E)}$, and negatively related to density. Delayed regulation ($\hat{\beta_{2}}$) was not related to climate, but to density (even though the explanatory power of both models was poor). The coefficients for the effect of climate ($\hat{\omega_{1}}$) were highest in poor and relatively stable climatic conditions, and unaffected by density.

### 3.2 Skew‐normally distributed environments (SKEW)

Surprisingly, $\hat{R}$ increased along the beneficial‐detrimental environmental gradient as it was positively related to environmental skewness ($\hat{\alpha }$), but as expected $\hat{R}$ was negatively related to population density ($\hat{D}$; Appendix S4: Fig. S4.6a). Both the $\hat{a_{R}}$ and $\hat{\gamma_{R}}$ also increased along the beneficial‐detrimental environmental gradient, and both were subject to negative density dependence (Appendix S4: Fig. S4.6b,d). As in the NORMAL simulations, density and climate were confounded (Figure 3b). Nonetheless, the DD model, which explained the most of the variance in $\hat{R}$, showed a negative and *a priori* expected pattern, whereas the DI model, which showed unexpected results, provided relatively less explanatory power. The reproductive allocation per unit female spring body mass $\hat{b_{R}}$ decreased along the beneficial‐detrimental gradient, that is, showed negative relationships with $\hat{\alpha }$, and increased as density increased (Appendix S4: Fig. S4.6c).

**Figure 4:**
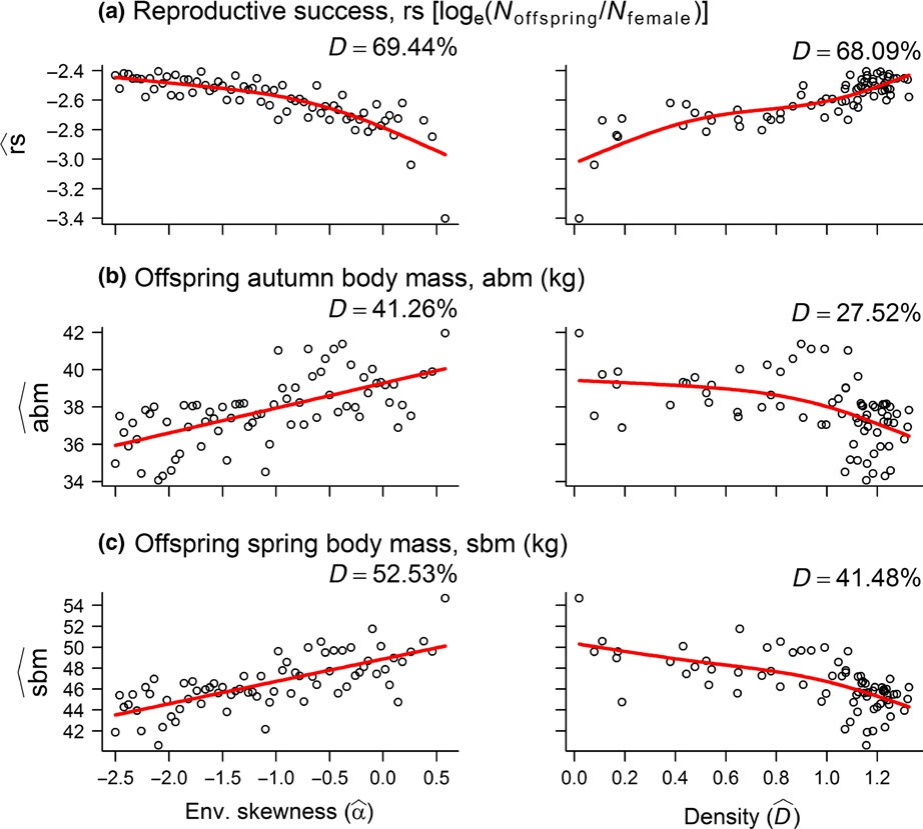
Generalized Additive Models (GAMs) showing how (a) reproductive success as well as (b) autumn and (c) spring offspring body mass was a function of environmental skewness ($\hat{\alpha }$, left panel) and population density ($\hat{D}$, right panel; GAM output provided in Appendix S4). Zero values for $\hat{\alpha }$ represent the standard normal distribution and represent a baseline for comparison, whereas a negative $\hat{\alpha }$ gives a skew toward the left and similarly a positive $\hat{\alpha }$ gives a skew toward the right: This represents a gradient from beneficial‐detrimental climatic conditions (Figure 1c)

**Figure 5:**
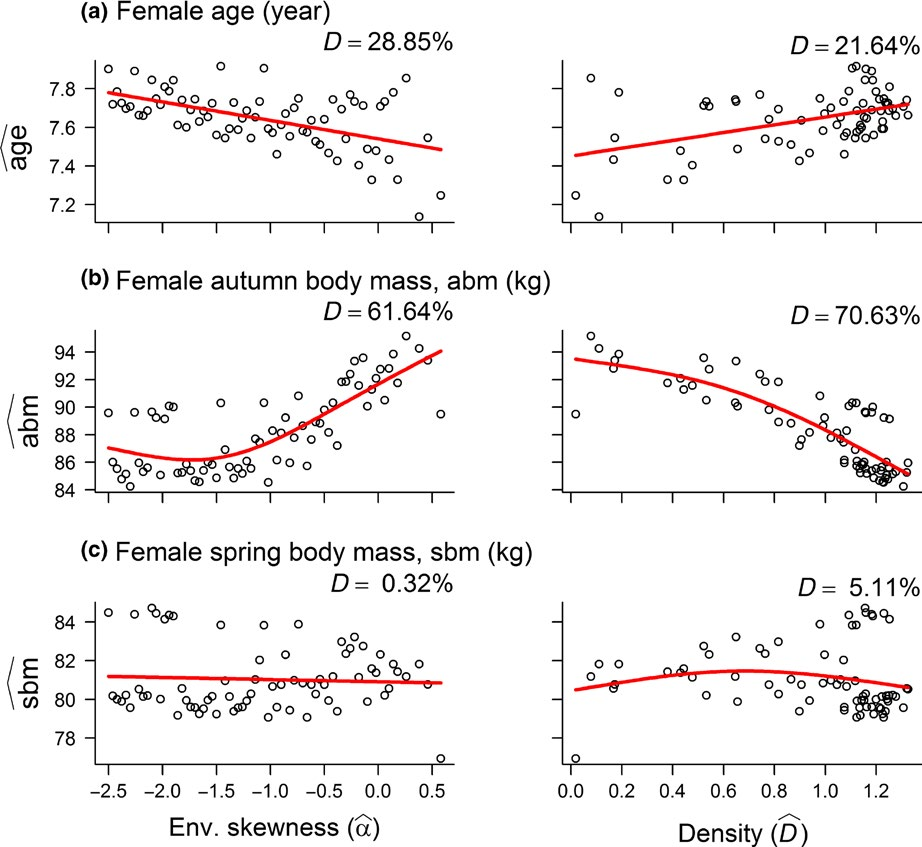
Generalized Additive Models (GAMs) showing how (a) adult age as well as (b) autumn and (c) spring adult body mass was a function of environmental skewness ($\hat{\alpha }$, left panel) and population density (D$\hat{\alpha }$, right panel; GAM output provided in Appendix S4)

As expected, $\hat{rs}$ decreased along the beneficial‐detrimental environmental gradient, meaning that the females allocated less to reproduction when the frequency of poor winter conditions increased, but its positive relationship with density was unexpected (Figure 4a). Offspring spring and autumn body mass increased along the beneficial‐detrimental environmental gradient and showed clear evidence of negative density dependence (Figure 4b,c). The female became younger along the beneficial‐detrimental environmental gradient, whereas age increased as density increased (Figure 5a). In the autumn, female body mass was positively related to $\hat{\alpha }$, and showed clear evidence of negative density dependence (Figure 5b). Spring body mass was, however, unaffected by both climate and density (Figure 5c).

**Figure 6:**
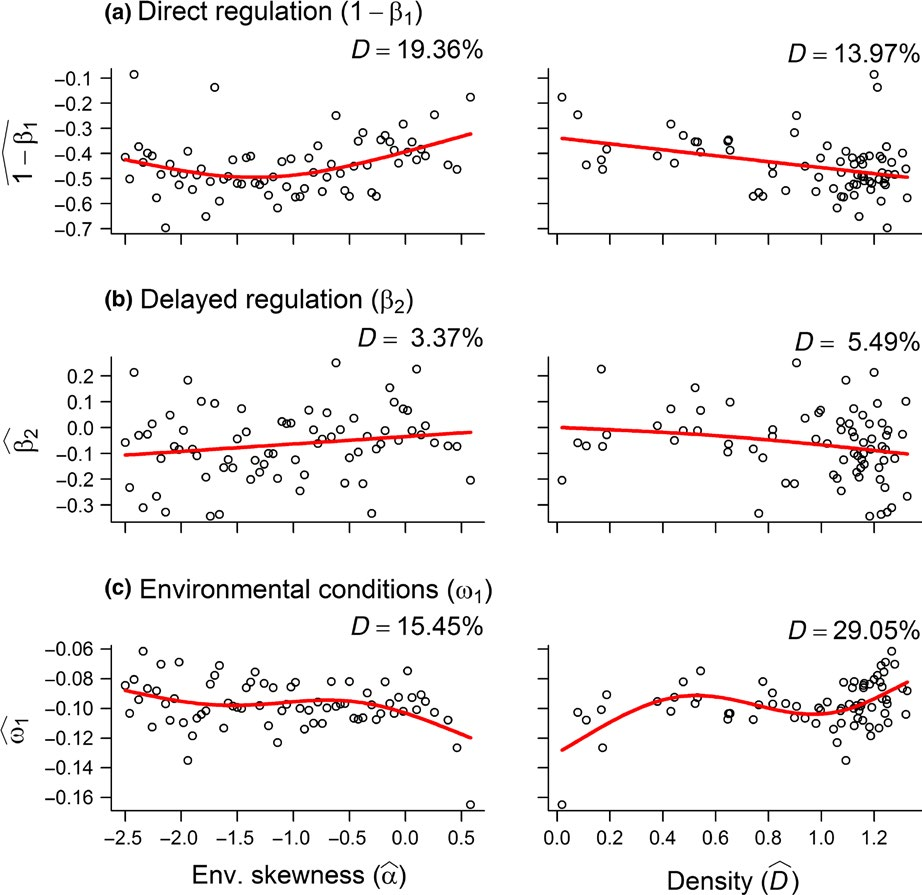
Generalized Additive Models (GAMs) showing how the estimated coefficient of the autoregressive model, that is (a) direct regulation (1 – β_1_), (b) delayed regulation (β_2_), and (c) direct effects of climate (ω_1_), was a function of environmental skewness ($\hat{\alpha }$, left panel) and population density ($\hat{D}$, right panel; GAM output provided in Appendix S4)

The effect of $\hat{1-\beta_{1}}$ showed a curved (concave‐down) relationship with $\hat{\alpha }$, being lowest at intermediate skewness, and a linear and negative relationship with density (Figure 6a). $\hat{\beta_{2}}$ was related to neither climate nor density (Figure 6b). The direct effect of climate ($\hat{\omega_{1}}$) on population growth was highest, that is, most negative, in beneficial environments, and increased as density increased (Figure 6c), indicating that the effect of climate on population growth interacted with density. Such an interaction was also supported by the fact that population density was explained by $\hat{\alpha }$ (Figure 3b): The highest densities were found in beneficial climates, whereas extinctions occurred when the frequency of poor climatic conditions reached a certain threshold (i.e., when $\hat{\alpha }\approx 0.5$).

**Figure 7:**
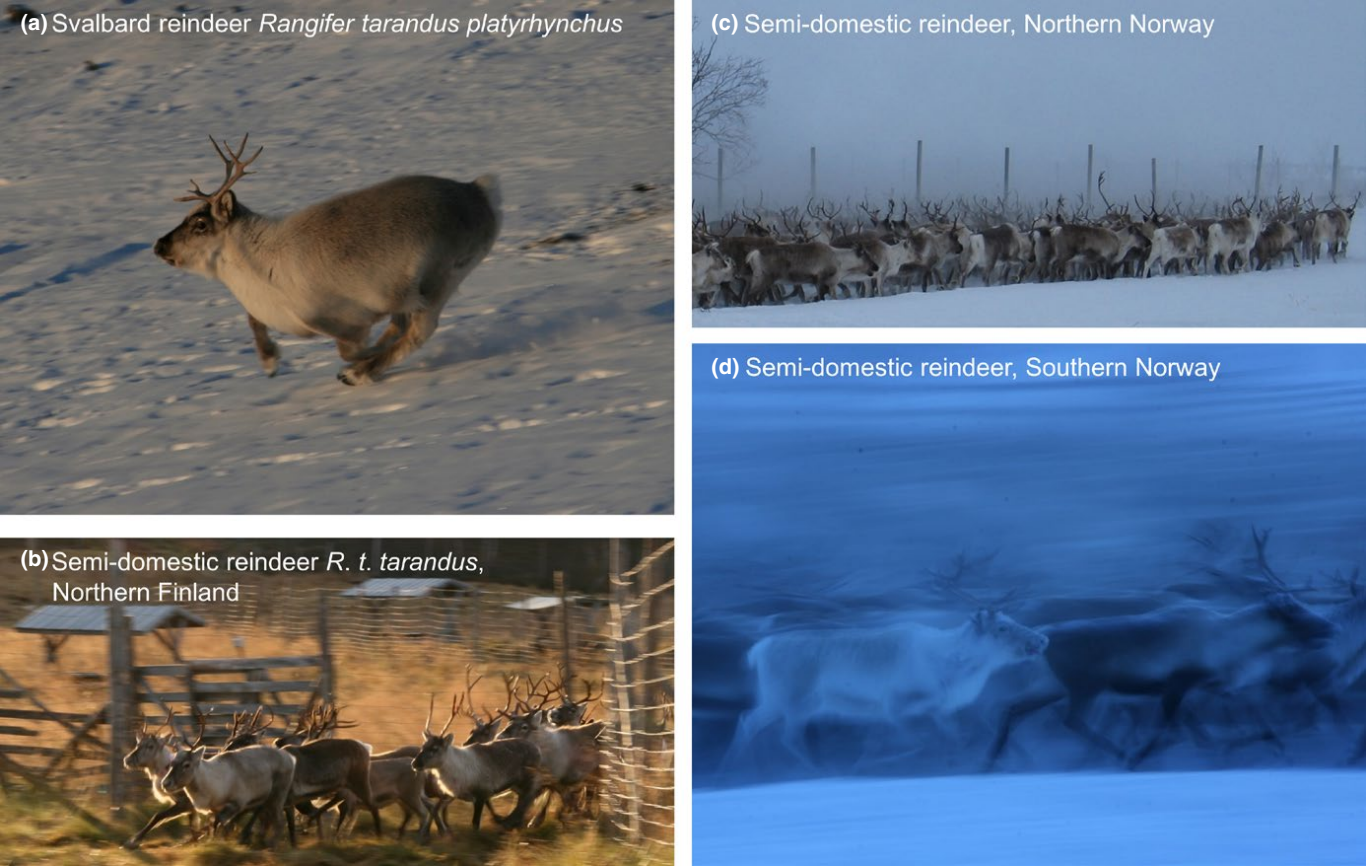
The present study uses a seasonal‐ and state‐dependent model showing that density dependent (DD) and density independent (DI) processes interact in affecting the evolution of reindeer reproductive strategies and population dynamics. The model, which was run using a wide range of different climatic scenarios, showed that the females have adopted a risk sensitive reproductive strategy. The picture shows two subspecies of reindeer experiencing different winter conditions. All photographs: B.‐J. Bårdsen

## 4 DISCUSSION

The present model predicts that life histories and population dynamics of a long‐lived mammal are affected by a combination of climatic conditions and population density. Female reproductive allocation, a result of different strategies, was subject to negative density dependence and seemed more affected by density than environmental conditions. Unexpectedly, reproductive allocation was highest in harsh (NORMAL) and detrimental (SKEW) environments (Figure 1 provided a schematic overview of the environmental gradients). The relative effect of density and climate, however, differed: (1) In the SKEW simulations, both the phenotype and its corresponding genotypic traits were better explained by the DD compared to the DI model; whereas (2) this was only the case for the phenotype and the body mass threshold in the NORMAL simulation. Reproductive success and female age were as expected low in harsh/detrimental environments, but increased as a function of density. Offspring autumn and spring body mass was highest in harsh/detrimental environments and subject to negative density dependence, but these findings were most apparent in the SKEW simulations. Population regulation interacted with climate while at the same time density and climatic conditions were confounded. Individuals responded, in line with a risk‐sensitive reproductive allocation, to climatic conditions and in a similar fashion across the two climatic conditions.

### 4.1 Individual life histories

#### 4.1.1 Reproductive strategies

Harsh winter conditions have for other herbivores previously been shown to: (1) increase mortality, notably for small individuals (e.g., Clutton‐Brock et al., 1996); (2) delay the onset of reproduction and lower reproductive effort (e.g., Sæther, Andersen, Hjeljord, & Heim, 1996; Sand, 1996); and 3) result in conditions where only the largest females are expected to reproduce (e.g., Festa‐Bianchet, Gaillard, & Jorgenson, 1998). Both the genotypic traits and their phenotypic expressions were affected by a combination of climate and density. Population density was confounded with climatic conditions as the most beneficial/benign climates supported the highest densities (Næss, Bårdsen, Pedersen, & Tveraa, 2011 discusses important system‐relevant confounders). This is an important yet complicating finding with respect to the interpretation of how individual respond to climate. The fact that females allocated slightly more to reproduction (*R*), and consequently less to survival (*S*), when climatic conditions were harsh/detrimental was unexpected (e.g., Adams, 2005), but can be explained by how density dependence was implemented through the gain function (Bårdsen et al., 2011: S1; Proaktor, Coulson, & Milner‐Gulland, 2007): density constrain the realized value a given allocation had on offspring (*R*) and female (*S*) autumn body mass. In sum, this means that density did not restrict allocation in the harsh/detrimental environments where density was low, as opposed to the high density found in the more beneficial/benign environments.

One shortcoming of the previous model was that individual strategies were modeled on an ordinal scale (Bårdsen et al., 2011:253), as in classic stochastic dynamic programming, because their genotypic traits could not change even though their phenotypic expression did. The present model rectified this as the three genotypic traits defining the phenotypes *simultaneously* evolved in response to environmental conditions, and on a continuous scale. Consequently, imposing less restrictions on how the strategies were allowed to evolve might mimic evolutionary processes more realistically and resulted in different values than the fixed values used for *a*_*R*_ and γ_*R*_ by Bårdsen et al. (2011:252–3; see also Appendix S5). This also had an impact on how environmental conditions affected female and offspring body mass and other empirically relevant measures. The present study also simulated increased frequency of extreme weather. This means that the present model closer mimics future scenarios for climate change (Appendix S1) and is more in accordance with how reindeer might adapt to such changes than the previous model.

The intercept and the body mass threshold showed similar relationships with both climate and density as reproductive allocation. The intercept, which represents a baseline value for reproductive allocation, was thus one factor leading to increased reproductive allocation in harsh/detrimental climatic conditions. The lower threshold body mass required for engaging in reproduction was, as expected, highest in the most severe environments and at high density—the smallest reproducing females were thus found in benign/beneficial climatic conditions and at high density. This is in line with earlier studies; female *Rangifer* need to reach a certain level of body reserves in order to reproduce (i.e., this effect is nonlinear: e.g., Skogland, 1985; Tveraa et al., 2003). The fact that females needed to grow larger to engage in reproduction in harsh/detrimental climates was thus expected. This together with the increased intercepts and low density in these environments seems to explain the unexpected relationship between reproductive allocation and climate.

The allocation per unit spring body mass (i.e., the slope) showed substantially higher variability than in Bårdsen et al. (2011:249) and was largest in beneficial/benign climates. This indicated that female allocated a smaller proportion of their spring reserves in harsh and detrimental climatic conditions (Appendix S4), which is in line with the abovementioned studies. This also means that reproductive allocation proportional to body mass was lowest in the harsh/detrimental environments.

#### 4.1.2 Empirical measures

Even though the relationship between climate and some of the theoretical measures presented above was unexpected, the more empirically relevant responses suggested that female reindeer have adopted risk‐sensitive life history (see details below). Additionally, *R* can be viewed as way of operationalizing the strategies, as it is not directly relevant to empirical studies, so the crucial test is to look at how the more empirically relevant model output (such a reproductive success and female autumn body mass: e.g., Bårdsen et al., 2008, 2010) responds to changing climatic conditions. Female body mass, that is, one of the key parameters affecting the theoretical quantities of allocation into reproduction (*R*) or survival (S) was higher in the most severe climatic conditions (due to reduced density dependence). This alone might explain the unexpected effect of climate on *R*, whereas more empirically relevant measures of reproductive allocation, such as reproductive success, were in accordance with expectations from the literature–that is, being lowest in harsh/detrimental environmental conditions.

Female autumn body mass was subject to negative density dependence—females experiencing high densities thus seemed unable to gain enough mass during summer to ensure own survival (e.g., Bårdsen & Tveraa, 2012; Skogland, 1985). Spring body mass was not largely affected by density, but was higher in good compared to poor environments (NORMAL simulation). This finding is similar to empirical studies where it has been suggested that females making strategic reproductive decisions during summer depending on how they experienced past winter conditions, whereas their winter body mass development is related to winter conditions (e.g., Bårdsen et al., 2008).

### 4.2 Population dynamics

The time series analysis revealed that population growth was affected by a combination of density and climatic conditions. First, in the NORMAL scenarios, direct density dependence occurred in all environments as $\hat{1-\beta_{1}}$ was always negative, but it was more pronounced in benign environments (see also Bårdsen et al., 2017 where direct regulation was more pronounced in the south compared to the north of Sweden). This was not surprising as density was high in these environments, and because interactions between density and winter conditions have previously been found for *Rangifer* (Ballesteros et al., 2013; Bårdsen & Tveraa, 2012, see also Tveraa et al., 2007). The delayed effect of density was strongest in good environments, but showed no relationship with climatic predictability. Population growth was more limited by climate in good relative to poor winter conditions, which, together with the abovementioned effects of climate on direct regulation, indicates that population dynamics was shaped by an interaction between density and climate (see also Tveraa et al., 2007 who found negative effects of climate for populations with access to good winter pastures).

Second, direct regulation occurred in all the SKEW simulations. But its concave‐up relationship with environmental skewness (Figure 4a) indicated that regulation was most pronounced at high frequencies of good and poor winters. As in the other simulations, the effect of climate was always negative, but more pronounced at low density. This provides additional support for the hypothesis that climate and density interact in shaping population growth, which is even further supported by the fact that extinctions occurred only in the most extreme environments (both simulations).

### 4.3 Risk sensitive life histories

Along with previous models and empirical studies, I document that long‐lived animals, such as reindeer, can adopt a risk‐sensitive reproductive allocation [reindeer (e.g., Bårdsen & Tveraa, 2012; Bårdsen et al., 2014); elk *Cervus elaphus* L. (Morano et al., 2012); white‐tailed deer *Odocoileus virginianus* Zimmermann (Simard et al., 2014); and mule deer *Odocoileus hemionus* Rafinesque (Monteith et al., 2013)]. “Risk sensitivity” as applied in the present context is adopted from economics, but biologists, anthropologists, and psychologists have for a long time recognized that the theory of economic allocation of a limited budget can be useful in studies of behavior (e.g., Kuznar & Frederick, 2003; Næss & Bårdsen, 2013; Winterhalder, 2007). Risk is defined as unpredictable variation in the outcome of behavior with consequences for an organism's fitness (the ultimate biological currency), utility (economic currency), or value (a synonym for both: e.g., Winterhalder, 2007), which can be viewed as the “prizes” that individuals—subject to particular constraints—seek to maximize.

Within behavioral ecology, risk sensitivity has its basis within optimal foraging theory even though it has been viewed as relevant for a wide range of different behaviors including reproduction (e.g., Bårdsen et al., 2014). Risk should be presumed important whenever the fitness function is nonlinear and one or more of the behavioral alternatives is characterized by unpredictable outcomes (e.g., Kacelnik & Bateson, 1996; Winterhalder, 2007). Risk is thus probably relevant for many organisms that have been classified as conservative, prudent, or selfish because the relationship between environmental conditions and important population vital rates (and hence also fitness) is often nonlinear (e.g., Henden, Bårdsen, Yoccoz, & Ims, 2008). Such nonlinear relationships—inducing an asymmetry between improved and worsened conditions—represents a problem of risk because individuals cannot manipulate the probability of encountering a harsh winter, but may buffer its adverse consequences by changing their behavior (e.g., going from a “risk‐prone” to “risk‐averse” reproductive allocation). Risk sensitivity thus expands on the traditional classification of long‐lived animal as conservative because individuals are expected to allocate fewer resources to reproduction and more to their own survival in harsh or unpredictable environments. Pertinently, risk sensitivity has predictive power as it predicts when and to what extent individuals should be risk‐averse (conservative) or risk‐prone (less conservative).

### 4.4 Future prospects

In poorly regulated populations, which are likely to reach numbers far above their carrying capacity, it has been suggested that negative climatic effects are strengthened at high density and reduced at lower density (e.g., Coulson et al., 2001; Milner, Elston, & Albon, 1999). For northern large herbivores, density may interact with winter climate in influencing body mass, which in turn can affect demographic traits such as survival (e.g., Coulson et al., 2001; Tveraa et al., 2003). Neither harvest nor predation was assessed in the present model, but both the simulated environments showed that reindeer population dynamics was formed by an interaction between the animals’ reproductive strategies and climatic conditions. The lack of regulation through harvest and predation is, however, a limitation likely to have major impact on the results (Appendix S5).

In fact, new research indicates that the removal of apex predators has resulted in dramatic ecosystem changes worldwide, which again influence how other stressors such as climate, habitat loss, and pollution affects these ecosystems (e.g., Estes et al., 2011). In northern Fennoscandia, for example, high reindeer abundance results in smaller animals that are more vulnerable to unfavorable conditions (Bårdsen & Tveraa, 2012; Tveraa et al., 2007) as they are most likely to die anyway (e.g., Tveraa et al., 2003). Predators typically target small, young, and weakened individuals while human slaughter strategies comes in many forms depending on the way animals are selected for harvest (e.g., Næss, Bårdsen, & Tveraa, 2012; Proaktor et al., 2007). The evolutionary impacts of predation and harvest might thus dramatically differ. Removal of smaller individuals, typical for predators, can impose a selective pressure in favor of delayed maturity and larger‐sized individuals (see Olsen et al., 2004; e.g., from fisheries). In contrast, selection of larger individuals, typically for trophy hunting, is known to have the opposite effect as it may result in smaller individuals and earlier maturation (reviewed by Milner, Nilsen, & Andreassen, 2007). A predator‐induced selection of smaller individuals is expected to positively affect the reindeer's ability to buffer negative climatic events as this leads to lowered density at the same time as the individuals being removed are likely to die anyway. The effect of selecting larger animals is, however, more uncertain as this will lower density at the same time as the harvested individuals are the ones with the highest survival and reproductive rates. This should be assessed in more details in future models.
