# Supplementary material for: Evolutionary responses to a changing climate: Implications for reindeer population viability
Source: Ecol Evol. 2017 Jun 20;7(15):5833–44. doi: 10.1002/ece3.3119 (PMC5551091; doi:10.1002/ece3.3119)
Supplement: Supplementary file 1 [file ECE3-7-5833-s001.pdf]

Appendix S1: Modelling Environmental Conditions

BACKGROUND

Winter climate was modelled on a relative scale where 'less is better' in the sense that large positive values represents poor conditions. This means that simulated environmental conditions has a similar effects as has been observed for climatic indices, e.g. the Arctic Oscillation Index (AO) and the North Atlantic Oscillation Index (NAO), on large herbivores in northern ecosystems (e.g. Aanes *et al.*, 2002, Helle & Kojola, 2008). Environmental conditions were simulated assuming two different statistical distributions (representing different climatic scenarios): 1) a normally distributed environment where I tested the effect of changing both the average and the variance (standard deviation); and 2) a skew normally distributed environment where I tested the effect of increasing the frequency of poor and good years (Fig. S1.1). In the latter scenario, parameter values were chosen as to increase the frequency of good and poor environments without changing the range in environmental conditions as defined in the simulation representing current environmental conditions. The latter is important as range in the observed values in the simulated skew-normal distributions generated was not allowed to exceed the extreme values gained in the standard normal distribution; $E \approx N(0,1)$.

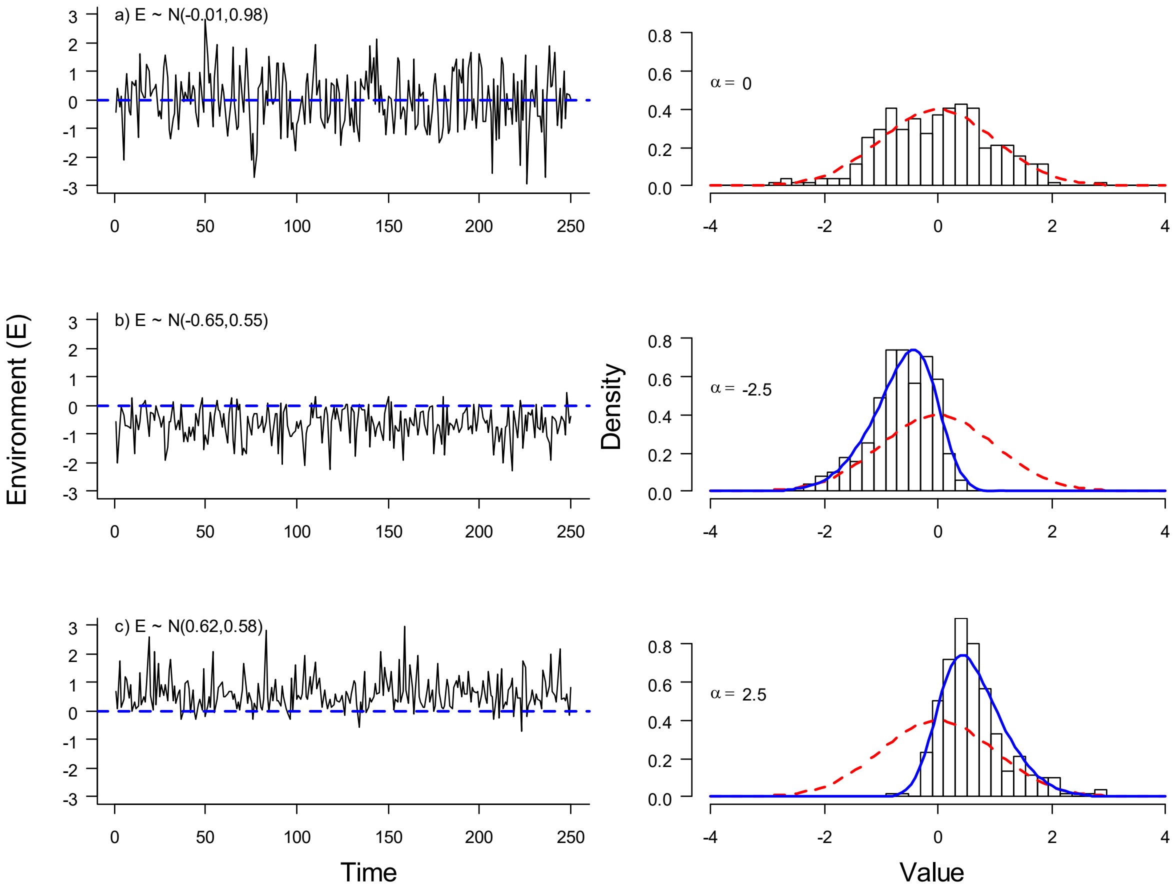

Fig. S1.1. Simulated environmental conditions (E) for 250 years for environmental conditions generated from a: (a) standard normal distribution [$E \approx N(0,1)$]: (b) skew-normal distribution with a shape parameter of -2.5 [$E \approx SN(\varepsilon, \omega^2, -2.5)$]; and (c) skew-normal distribution with a shape parameter of 2.5. **Blue** lines in the right panel show the probability density for the skew-normal distributions, whereas the **red** lines show the probability density for the standard normal distribution.

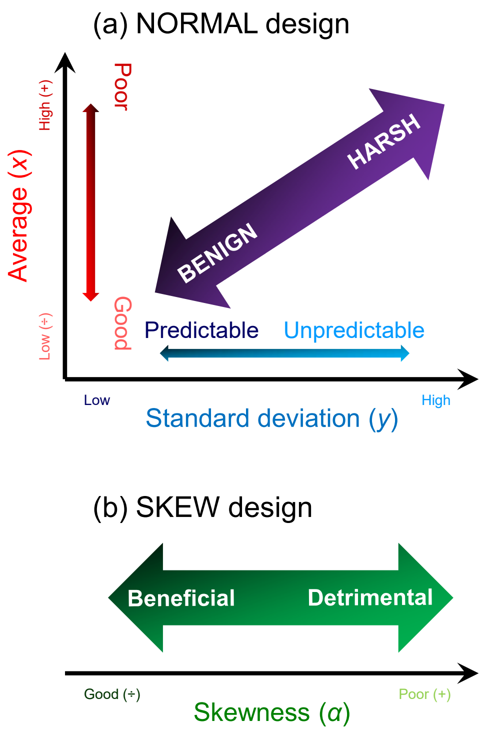

Fig. S1.2. Conceptual figure showing the gradients in climatic conditions for simulations consisting of normally distributed (NORMAL, a) and skew-normally distributed (SKEW, b) environmental conditions.

The SKEW simulations are in line with the predictions from the literature: future global climate change will most likely result in a shift towards more frequent extreme precipitation events (e.g. Wilby & Wigley, 2002, Semmler & Jacob, 2004, Tebaldi *et al.*, 2006, Benestad, 2007, Sun *et al.*, 2007), a trend that is already empirically evident on several continents (Sun *et al.*, 2007 and references therein). Hanssen-Bauer *et al.* (2005), for example, review several studies predicting how climate will change in Fennoscandia in the future (see also Benestad, 2011): 1) increased warming rates with distance to the coast; 2) higher warming rates in winter compared to summer; and 3) increased precipitation especially during winter. The shifts between warm and cold periods during winter coupled with an year-round increased intensity of precipitation (Hanssen-Bauer *et al.*, 2005), will lead to an increased frequency of wet weather, deep snow and ice crust formation that has negative consequences for large herbivores (e.g. Solberg *et al.*, 2001).

Rising temperatures and changing precipitation patterns have already been suggested to lead to population declines for *Rangifer* (Vors & Boyce, 2009). In the European Arctic and Sub-Arctic, increased occurrences of rain-on-snow and freeze-thaw events have had negative impacts on reindeer demography and population growth (Hansen *et al.*, 2011, Solberg *et al.*, 2001), and these are candidates for causing more frequent population collapses (see also Pape & Löffler, 2012). Yet, not all the predicted changes have to be negative (Pape & Löffler, 2012:Table 3). For semi-domestic reindeer in Europe, pasture quality (earlier spring, longer growing season and higher biomass of edible plants) combined with climate change are believed to have negative effects in Scandinavia (Sweden and Norway), to be neutral in Finland and to have positive effects in Russia (Rees *et al.*, 2008). It is thus, uncertain if the overall effect of climate change will be positive or negative, or, alternatively neutral.

NORMAL DISTRIBUTION (NORMAL simulations)

For these simulations, climatic conditions were simulated as white noise characterized by a given average (x) and a standard deviation (y): [$E \approx N(x,y)$]. The three averages in the middle (i.e. $x = -0.15$, 0 and 0.15; see main text for details) represented the same environments as in Bårdsen *et al.* (2011), whereas two more extreme environments has been added in the present study (i.e. $x = -0.30$, and 0.30). The standard normally distributed environment, i.e. $E \approx N(0,1)$, may be viewed as a reference as this resents the distribution currently observed for the AO (see Appendix A1 in Bårdsen *et al.*, 2011 for details). For this distribution there exists three continuous climatic gradients going from (Fig. S1.2a): 1) good to poor (defined as increased x); 2) predictable to unpredictable (defined as increased y); and 3) benign to harsh (defined as the simultaneous increase in x and y).

SKEW-NORMALLY DISTRIBUTION (SKEW simulations)

For these simulations, climatic conditions were simulated as a skew-normal distribution [$E \approx SN(\varepsilon, \omega^2, \alpha)$], where ε, ω and α represent the location, scale and the shape parameters (Azzalini,

2005, Azzalini, 2007). The shape parameter controls the degree of skewness: negative or positive values indicate skew to the left or right, and as these values increase towards -∞ or ∞ the probability density converges towards a half-normal distribution, whereas zero values give the standard normal distribution [i.e. $\approx N(\varepsilon, \omega^2)$]. I manipulated this distribution keeping the location and scale parameters constant at 0 and 0.85, whereas the shape parameter varied as follows: $\alpha = [-2.5, -2.46, -2.42 \ldots, 2.5]$, which resulted in 126 simulated environments (see Fig. S1.1. for an example showing the most extreme environments in comparison to the standard normal distribution). For this distribution, there exists one continuous climatic gradient (Fig. S1.2b): going from beneficial (high frequency of good conditions) to detrimental (high frequency of poor conditions; defined as increased α).

REFERENCES

Aanes, R., Sæther, B.-E., Smith, F.M., Cooper, E.J., Wookey, P.A. & Øritsland, N.A. (2002) The Arctic Oscillation predicts effects of climate change in two trophic levels in a high-arctic ecosystem. Ecology Letters, **5**, 445-453.

Azzalini, A. (2005) The skew-normal distribution and related multivariate families. Scandinavian Journal of Statistics, **32**, 159-188.

Azzalini, A. (2007) The sn package: the skew-normal and skew-t distributions. R package version 0.4-4. University of Padova, Deptartment of Statistical Sciences, Padova, Italy.

Benestad, R.E. (2007) Novel methods for inferring future changes in extreme rainfall over Northern Europe. Climate Research, **34**, 195-210.

Benestad, R.E. (2011) A new global set of downscaled temperature scenarios. Journal of Climate, **24**, 2080-2098.

Bårdsen, B.-J., Henden, J.-A., Fauchald, P., Tveraa, T. & Stien, A. (2011) Plastic reproductive allocation as a buffer against environmental stochasticity - linking life history and population dynamics to climate. Oikos, **20**, 245-257.

Hansen, B. B., Aanes, R., Herfindal, I., Kohler, J. & Sæther, B. E. (2011). Climate, icing, and wild arctic reindeer: past relationships and future prospects. Ecology **92**, 1917-1923.

Hanssen-Bauer, I., Achberger, C., Benestad, R.E., Chen, D. & Førland, E.J. (2005) Statistical downscaling of climate scenarios over Scandinavia. Climate Research, **29**, 255-268.

Helle, T. & Kojola, I. (2008) Demographics in an alpine reindeer herd: effects of density and winter weather. Ecography, **31,** 221-230.

Pape R. & Löffler J. (2012) Climate change, land use conflicts, predation and ecological degradation as challenges for reindeer husbandry in Northern Europe: what do we really know after half a century of research? Ambio **41**,421-434.

Rees W., Stammler F., Danks F. & Vitebsky P. (2008) Vulnerability of European reindeer husbandry to global change. Climatic Change **87**,199-217

Semmler, T. & Jacob, D. (2004) Modeling extreme precipitation events - a climate change simulation for Europe. Global and Planetary Change, **44**, 119-127.

Solberg, E.J., Jordhøy, P., Strand, O., Aanes, R., Loison, A., Sæther, B.E. & Linnell, J.D.C. (2001) Effects of density-dependence and climate on the dynamics of a Svalbard reindeer population. Ecography, **24**, 441-451.

Sun, Y., Solomon, S., Dai, A.G. & Portmann, R.W. (2007) How often will it rain? Journal of Climate, **20**, 4801-4818.

Tebaldi, C., Hayhoe, K., Arblaster, J.M. & Meehl, G.A. (2006) Going to the extremes. Climatic Change, **79**, 185-211.

Vors, L.S. & Boyce, M.S. (2009). Global declines of caribou and reindeer. Global Change Biology, **15**, 2626-2633.

Wilby, R.L. & Wigley, T.M.L. (2002) Future changes in the distribution of daily precipitation totals across North America. Geophysical Research Letters, **29**.

Appendix S2: Model Initiation

BACKGROUND

I initiated all simulations with the same population, i.e. using the exact same individuals, in order to keep initial conditions constant across the simulations. The model was initiated at $t = t_0$ by creating 200 individuals (n_{t_0}), which resembles a population density of 0.40 individuals km^{-2}, with a constant age of 2 years:

(1) Spring body mass was a random number drawn from a normal distribution with a given average (x) of 60.7 kg and a standard deviation (y) of 5.0 kg [i.e. $\approx N(x, y)$].

(2) Similarly, the threshold spring body mass for reproduction was generated as $\gamma_{R_{t_0}} \approx N(43.2, 3.0)$.

(3) Then, the population was divided into two equally sized subsets ($Data_a$ and $Data_b$).

(4) $Genotype_{Range_a}$ and $Genotype_{Range_b}$, which substitutes the range in the deterministic values for a_R or b_R, respectively, were defined as follows: $Genotype_{Range_a} \in [min(Genotype_{Range_a}), \ldots, max(Genotype_{Range_a})]$ added to $Data_a$; and $Genotype_{Range_b} \in [max(Genotype_{Range_b}), \ldots, min(Genotype_{Range_b})]$ added to $Data_b$ (the steps between the minimum and maximum was equally spaced).

(5) Finally, $Genotype_{a_{t_0}}$ and $Genotype_{b_{t_0}}$, which substitutes the realized values for a_R and b_R at t_0, was created by adding a normally distributed error to these deterministic values: For a_R, $Genotype_{a_{t_0}} = Genotype_{Range_a} + Initial_{error_a}$ (defined as $Initial_{error_a} \approx N(0, Initial_{SD_a})$; and for b_R $Genotype_{b_{t_0}} = Genotype_{Range_b} + Initial_{error_b}$ (defined as $Initial_{error_b} \approx N(0, Initial_{SD_b})$.

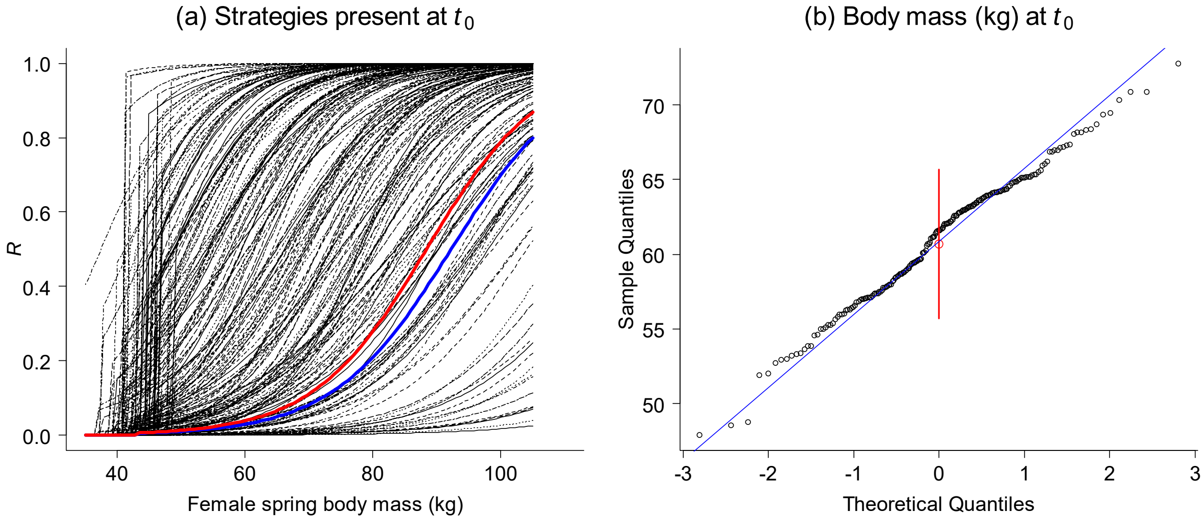

Figure S2.1. (a) Between-individual variability in strategies, where each line represents one female, present in the population when the simulation was initiated (i.e. at $t = t_0$). The thick lines in **blue** and **red** shows the two most winning strategies in the previous study (Bårdsen *et al.*, 2011). (b) Normal quantile-quantile (QQ) plots for initial spring body mass. The point and line shows the given average and standard deviation used for generating the body masses.

At first glance, this might be viewed as unnecessary complex, but the point of this exercise was to ensure a high degree of between-individual variability during model-initialization with respect to the parameters defining the strategies (Fig. S1.1a) and body mass (Fig. S1.1b). This was assured by combining different values for the three genotypic traits (the deterministic part of the initiation; point 3-4 above) while inducing some randomization to this process (point 5 above; Table S2.1 provides information about the parameter used).

Table S2.1. Values used for the initiation of the model.

Parameter	Explanation	Value (scale)	Source/Notes
t_0	Initial time (spring)	0 (year)	———
A	Study area	500 (km^2)	———
n_{t_0}	Number of females present at t_0	200 (number)	———
j_{t_0}	Initial age for all individuals	2 (year)	(Bårdsen *et al.*, 2011)
j_{max}	Maximum (max) possible age	16 (year)	(Bårdsen *et al*, 2011)
$Spring_{bm_{t_0}}$	Initial spring body mass was generated from a normal distribution with a given mean (x) and standard deviation (y) [i.e. $\approx N(x, y)$]	x = 60.70 (kg)<br>y = 5.00 (kg)	(Bårdsen *et al.*, 2011)
$\gamma_{R_{t_0}}$	Reproductive spring body mass threshold was generated from a normal distribution with a given mean (x) and standard deviation	x = 43.20 (kg)<br>y = 3.00 (kg)	(Bårdsen *et al.*, 2011)
$Genotype_{Range_a}$	Range (minimum and maximum) in intercept values (a_R)	-10.000,-7.000	———
$Genotype_{Range_a}$	Range in slope values (b_R)	0.080,0.170	
$Initial_{SD_a}$	Standard deviation for the error added to the deterministic intercept values (a_R)	2.000	———
$Initial_{SD_b}$	Standard deviation for the error added to the deterministic intercept values (b_R)	0.001	

REFERENCES

Bårdsen, B.-J., Henden, J.-A., Fauchald, P., Tveraa, T. & Stien, A. (2011) Plastic reproductive allocation as a buffer against environmental stochasticity - linking life history and population dynamics to climate. Oikos*,* **20**, 245-257.

Appendix S3: Running and Interpreting the Model

RUNNING THE MODEL

Design and initiation

At t_0, 200 individuals was generated with a constant age, normally distributed body mass, and ensuring randomness while at the same time a high degree of between-individual variability with respect to all three genotypic traits (Appendix S2). Simulations, statistical tests and plotting was performed in *R* (R Core Team, 2013).

Winter climatic conditions

I ran the model with two sets of winter climatic conditions (E). I modelled winter climate on a relative scale where 'less is better' in the sense that large positive values represent poor conditions (details provided in Appendix S1).

Climatic scenario I: normally distributed environments (NORMAL)

First, similar to Bårdsen *et al.* (2011), E was modelled as white noise characterized by a given average (x) and standard deviation (y): [$E \approx N(x, y)$]. Five different average climatic regimes ($x = [-0.30, -0.15, 0, 0.15, 0.30]$) was then applied (the two most extreme values were not used in Bårdsen *et al.*, 2011). Different levels of climatic variability was added to each average condition ($y = [0.04, 0.08, 0.12, \ldots, 2.00]$), and this resulted in a total of 250 possible environments ($x \times y$). Nonetheless, due to extinction before convergence in the most extreme environments, i.e. those with high values for y and/or x, the total number of simulations was only 147 (i.e. extinction occurred in ~40 % of the simulations). For this distribution there exists three continuous climatic gradients going from (see Appendix S1): (1) *good* to *poor* (defined as increasing x); (2) *predictable* to *unpredictable* (defined as increasing y); and (3) *benign* to *harsh* (defined as a simultaneous increase in x and y).

Climatic scenario II: skew-normally distributed environments (SKEW)

E was modelled as a skew-normal distribution in order to simulate an increased frequency of both poor and good environments, through varying the shape parameter (hereafter referred to as environmental skewness: $\alpha = [-2.5, -2.46, -2.42 \ \ldots, 2.5]$, see Appendix S1 for details), which resulted in a total of 126 potential simulations. For this distribution there exists only one continuous climatic gradient going from *beneficial* (high frequency of *good* conditions) to *detrimental* [high frequency of *poor* conditions; defined as increased skewness values (α; Appendix S1)]. Extinction before convergence happened in the most extreme environments (the most extreme positive α with live population was 0.58). Only 72 simulations contained live populations in the end so similar to the NORMAL simulations extinction occurred in ~40 % of the runs. When $\alpha = 0$ this resembles the standard normal distribution, i.e. $N(0,1)$, which was the only simulation directly comparable to any of the NORMAL-scenarios. When $\alpha < 0$ and $\alpha > 0$ the distribution of the simulated environments were skewed towards negative and positive values, respectively. This, however, also changed the mean and the variability of the distribution (Appendix S1).

Convergence

In the previous model convergence was reached when only one strategy was left in the population. Because strategies were allowed to evolve continuously in the present model, assessing convergence became more difficult. Natural selection is a process that leads to traits becoming more or less common in a population, which means that natural selection affect the distribution of traits within a population over time (e.g. Coulson *et al.*, 2006). Consequently,

convergence was achieved when evolution had stabilized the distribution of the three genotypic traits defining the reproductive strategies. I thus assumed convergence to be achieved when the variability of all genotypic traits showed no temporal trends even though they were allowed to vary both between- and within-years. Convergence was assessed within a subjectively defined time window (t_{conv}) set to 75 years, and convergence was achieved when no significant temporal trends in any of the traits occurred within this time window. Convergence was thus assessed iteratively for each time step (t) in a simulation. As convergence cannot be achieved when $t \leq t_{conv}$, convergence for genotypic trait i (where i can be substituted by a_R, b_R and γ_R; see eqn. 1-2 in the main text) was assessed and updated as follows when $t > t_{conv}$:

(1) the year-specific coefficient of variation, i.e. the estimated standard deviation divided by the estimated average, was calculated for each trait [$cv(Genotype_i)_t$];
(2) for a given time step (t') in a simulation, the values for $cv(Genotype_i)_t$ from $t = t' - t_{conv}$ to $t = t'$ was extracted;
(3) the residuals from a linear regression model fitting only the intercept was fitted using $cv(Genotype_i)_t$ as the response [in the software R: lm($cv(Genotype_i)_t$~1)];
(4) any significant, positive or negative, correlations (using the cor.test function in R) between the residuals and the specified time period revealed that the model had not converged with respect to genotypic trait i (when convergence was achieved, i.e. when this correlation was non-significant, no further testing was performed); and
(5) the simulation converged when no such significant correlation was apparent for any of the three genotypic traits.

Strictly speaking, I assessed convergence as a lack of any temporal trends in the variance of each genotypic trait, but a visual inspection of the annual averages and medians also indicates a lack of any temporal trends for these statistics. To show details about this I checked the details for a selection of environments: 1) the standard normal environment (Fig S3.1); 2) the most benign environment (Fig S3.2); and 3) the harshest environment (Fig. S3.3).

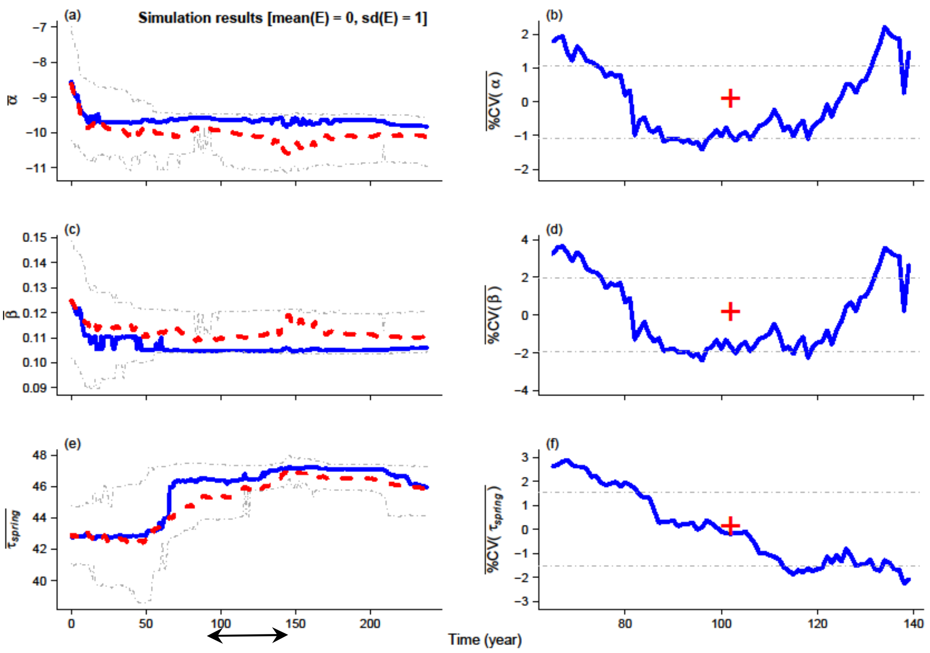

Fig. S3.1. Assessing convergence for the simulation that represents the standard normal distribution, i.e. when $E \approx N(0,1)$. The left panel shows temporal trends for the median (blue), average (red dotted lines) as well as the 25th and 75th quartiles for the three genotypic traits [the intercept (a), slope (b) and body mass threshold (c)] for the whole simulations (the arrow indicates the period in which convergence was assessed). The right panel shows temporal trends

in the coefficient of variation (in percentages) for each genotypic trait for the 75 years before convergence. The left panel shows that even though there still was variability in the genotypic traits there was no apparent trends in the mean and average values after the point of convergence.

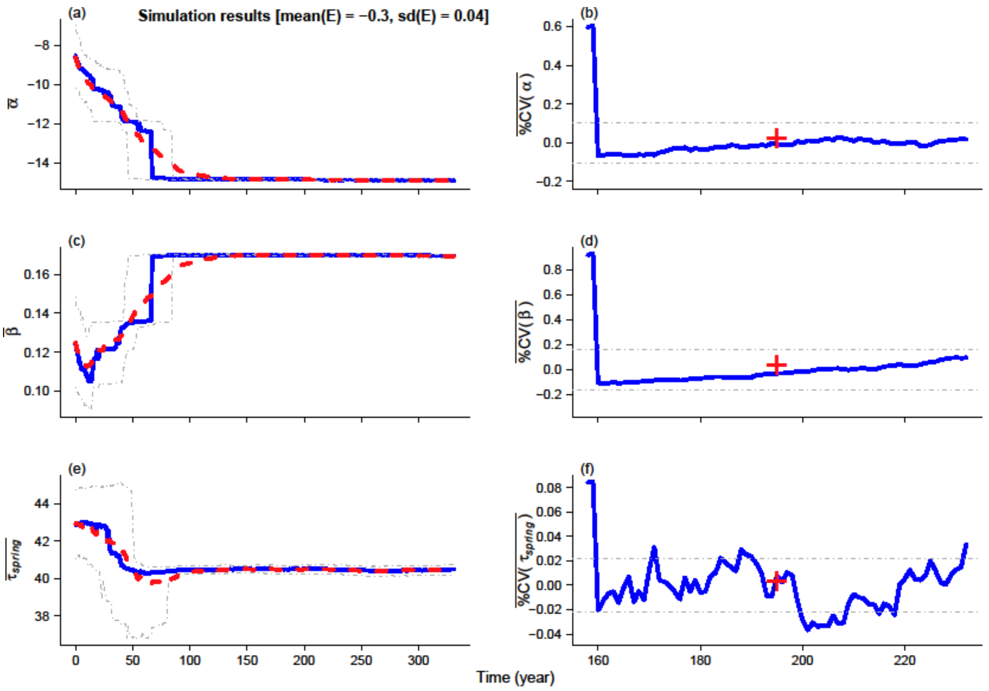

Fig. S3.2. Assessing convergence for the simulation that represents most benign environment, i.e. when $E \approx N(-0.3{,}0.04)$. Technical details presented in Fig. S3.1.

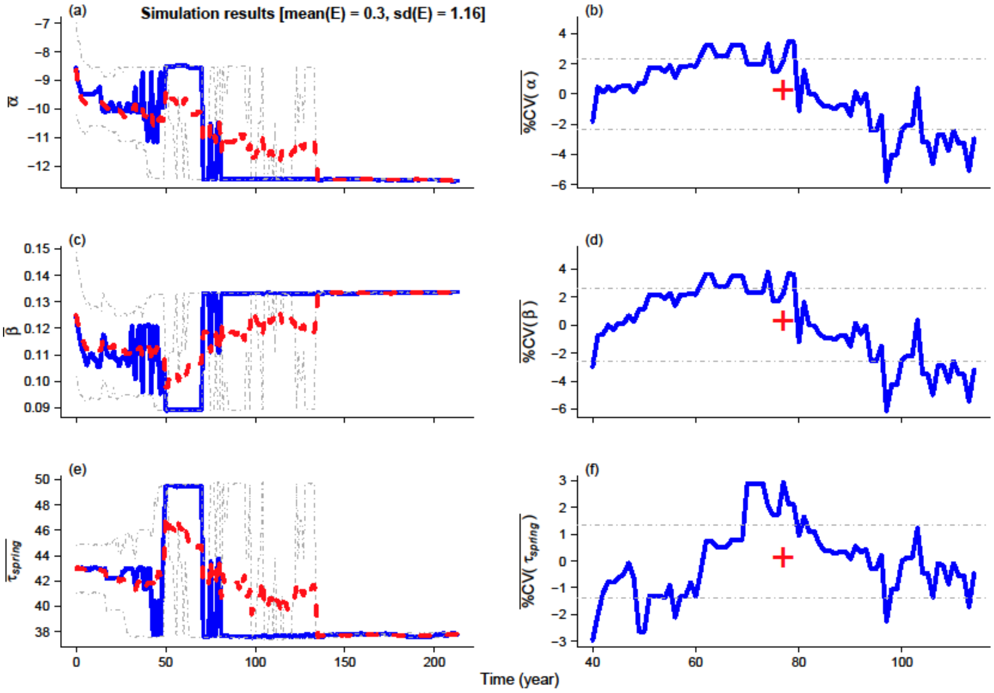

Fig. S3.3. Assessing convergence for the simulation that represents harshest environment, i.e. when $E \approx N(0.3{,}1.16)$. Technical details presented in Fig. S3.1.

ANALYZING AND INTERPRETING RESULTS

Responses and predictors

At the point of convergence the simulations ran for 200 more years (the data collection period) reaching terminal time (T) when the simulation was ended. The following annual population-level data was recorded: (1) across-year average density ($\widehat{D}$); and (2) winter climate conditions; across-year environmental average ($\hat{E}$) and its standard deviation [$\widehat{\text{sd}(E)}$] for the NORMAL simulations and skewness ($\hat{\alpha}$) for the SKEW simulations. Moreover, the average across all individuals and years of the following individual-level data was recorded across the whole data collection period: (1) the three genotypic traits ($\widehat{a_R}$, $\widehat{b_R}$ and $\widehat{\gamma_R}$) as well as the resulting phenotypic expression ($\hat{R}$); (2) female reproductive success ($\widehat{rs}$; offspring per female on $\log_e$-scale); (3) spring and autumn body mass ($\widehat{sbm}$ and $\widehat{abm}$) for females and offspring, respectively; and (4) female age ($\widehat{age}$). The utilization of values across years and individuals differ from the previous study (Bårdsen *et al.*, 2011:249).

PSEUDO-EMPIRICAL ANALYSES

Evolution of strategies

The analyses of the dynamic optimization assessed how reproductive allocation, and the three genotypic traits, was dependent on environmental conditions and density. In addition, I also analyzed more commonly used empirically used measures of individual life histories.

Allocation of resources to reproduction and survival

Generalized Additive Models (GAM), using the mgcv library (Wood, 2012), were fitted using estimated averages responses (see above) from a given simulation. The resulting dataset contained one data point for each response/predictor(s) from a given simulation. Thin plate regression splines were used to model potential non-linear effects of average population density ($\widehat{D}$) and the interaction between both environmental characteristics [i.e. the estimated climatic variability [$\widehat{\text{sd}(E)}$] and average climate ($\hat{E}$)] (Wood, 2006). The following code was applied in the R software: 1) the *Density Independent* (DI) model specified as 'gam(response ~ s($\widehat{\text{sd}(E)}$,$\hat{E}$, bs=tp, k=4)' and 'gam(response ~ s($\hat{\alpha}$, bs=tp, k=4)' for the NORMAL and SKEW simulation, respectively; and 2) the *Density Dependent* (DD) model specified as 'gam(response ~ s($\widehat{D}$, bs=tp, k=4)'. Plotting of results from the DI model in the NORMAL simulations was performed using the vis.gam function (as this model contained two continuous predictors), whereas plotting the DI and DD model in the remaining analyses were performed using the predict.gam function (Wood, 2012).

Population dynamics: time series analyses using autoregressive models

As in the previous study, I assessed to what extent climatically induced changes in life histories affect population dynamics, which were analyzed using time series analysis. The population growth rate, i.e. $\lambda_t = log_e(N_{t+1}) - log_e(N_t)$, where N_t refers to populating abundance in a given year (t), was modelled using second-order autoregressive models [AR(2), fitting an ARIMA(p = 2, d = 0, q = 0) model using the arima-function where $\hat{E}$ was included as a covariate in the xreg argument]. The linear predictor of the models included direct (t) and delayed ($t-1$) density dependence (regulation) and the direct effect of E_t on λ_t. I, thus, estimated the first-order AR coefficient ($1+\beta_1$), the second-order AR coefficient (β_2) and the direct effect of winter climate conditions (ω_1). Statistical analyses, using the resulting coefficients from the time series analyses as responses, and plotting was performed similarly as in the previous paragraph.

REFERENCES

Bårdsen, B.-J., Henden, J.-A., Fauchald, P., Tveraa, T. & Stien, A. (2011) Plastic reproductive allocation as a buffer against environmental stochasticity - linking life history and population dynamics to climate. Oikos, **20**, 245-257.

Coulson, T., Benton, T.G., Lundberg, P., Dall, S.R.X., Kendall, B.E. & Gaillard, J.M. (2006) Estimating individual contributions to population growth: evolutionary fitness in ecological time. Proceedings of the Royal Society B-Biological Sciences, **273**, 547-555.

R Core Team. (2013) R: a language and environment for statistical computing. R Foundation for Statistical Computing, Vienna, Austria.

Wood, S.N. (2006) Generalized additive models: an introduction with R. Chapman & Hall/CRC.

Wood, S.N. (2012) mgcv: GAMs with GCV/AIC/REML smoothness estimation and GAMMs by PQL. R package.

Appendix S4: Detailed Results

BACKGROUND

In this appendix I show more detailed results compared to what is shown in the main text.

RESULTS

Normally distributed environments (NORMAL)

Evolution of strategies

Reproductive allocation ($\hat{R}$), which is the strategies' phenotypic expression, was positively related to both environmental average ($\hat{E}$) and environmental variability [$\widehat{\mathrm{sd}(E)}$], and negative related to population density ($\hat{D}$; Table S4.1a & Fig. S4.1). This lead to the surprising result that female reindeer allocated more resources to reproduction when environmental conditions were harsh (i.e. in poor and unpredictable) environments. The range in the predicted values of $\hat{R}$ was, however, small while at the same time as the deviance explained was >20% higher for the density dependent (DD) model compared to the density independent (DI) model (Fig. S4.1). Additionally, population density was highest in benign (i.e. in good and predictable) environments and lowest in harsh environments (Fig. S4.2). The fact that climate was such a powerful predictor of density (Fig. S4.2) implies that density and climate was confounded – and hence their effects may be difficult, if not impossible, to disentangle in simulations where harvest or predation does not occur (see main text for details). The relative difference in the deviance explained for the DD model, however, indicates that density might be a more important predictor of $\hat{R}$ than climate, which showed the pattern that I *a priori* expected. The intercept ($\widehat{a_R}$) and the body mass threshold ($\widehat{\gamma_R}$; the lowest spring body mass the females' needed for engaging in reproduction), i.e. two of the strategies' genotypic traits, showed, similar to $\hat{R}$, positive relationships with $\hat{E}$ and $\widehat{\mathrm{sd}(E)}$, and both were subject to negative density dependence (Table S4.2b,d & Fig. S4.1b,d). Judging from the percentage deviance explained, $\widehat{a_R}$ was more affected by climate than density, whereas this was the opposite for body mass threshold. The body mass threshold was, however, poorly explained by both the DD and DI models. The slope for spring body mass ($\widehat{b_R}$), which was the only genotypic trait being optimized in the previous study, showed negative relationships with both $\hat{E}$ and $\widehat{\mathrm{sd}(E)}$, and a positive relationship with density (Table S4.1c & Fig. S4.1c).

Reproductive allocation: reproductive success and offspring body mass

By using measures of reproduction commonly used in empirically studies, I attempted to relate model output to the empirical world. Reproductive success ($\widehat{rs}$: the number of offspring per female on $\log_e$-scale) was highest in benign and lowest in harsh environments [i.e. negatively related to both $\hat{E}$ and $\widehat{\mathrm{sd}(E)}$]. This was expected, but it was the opposite what I found for $\hat{R}$. Females also produced more offspring in high- vs. low-density environments (Fig. S4.3a & Table S4.2a), but such a positive density dependence was unexpected. Again, this could be because density and climate was confounded: for $\widehat{rs}$, the DI model the deviance explained was more than three-fold compared to the DD model. This might indicate that the relationship between $\widehat{rs}$ and climate, which supported my *a priori* expectations, was stronger than the relationship between $\widehat{rs}$ and density (Fig. S4.3a). It is, however, important to note that the range in the predicted values for $\widehat{rs}$ was low. Offspring spring and autumn body mass was highest in harsh and lowest in benign environments, but showed evidence of negative density dependence (Fig. S4.3b,c & Table S4.2b,c).

Somatic allocation: female age and body mass

Female age was highest in benign and lowest in harsh environments, whereas females became older as density increased (Fig. S4.4a & Table S4.3a). In the autumn, females were largest in harsh environments, and showed clear evidence of negative density dependence (Fig. S4.4b & Table

S4.3b). In spring, however, females were smallest in poor and predictable environments and largest in good and unpredictable environments, but showed no evidence for density dependence (Fig. S4.4c & Table S4.3c).

Population dynamics: time series analyses
The effect of direct regulation ($\widehat{1-\beta_1}$) was most pronounced, i.e. the most negative, in harsh environments and negatively related to density (Fig. S4.5a & Table S4.4a). This means that direct negative density dependence increased, i.e. became more negative, along the benign-harsh gradient and as density increased. Delayed regulation ($\widehat{\beta_2}$) was not significantly related to climate, but were negatively related to density (even though the explanatory power of both these model were poor: Fig. S4.5b & Table S4.4b). The direct effect of climate ($\widehat{\omega_1}$) on population growth was highest in poor and relatively stable climatic conditions, and unaffected by density (Fig. S4.5c & Table S4.4c).

Skew-normally distributed environments (SKEW)
Evolution of strategies
Unexpectedly, reproductive allocation ($\hat{R}$) was positively related to the environmental skewness (α), which means that females allocated more to reproduction when the frequency of poor winter conditions increased (i.e. under detrimental conditions: Appendix S1), and negatively related to density ($\hat{D}$): Table S4.5a & Fig. S4.6a]. The fact that $\hat{R}$ increased along the beneficial-detrimental gradient could, similar to the findings for the normally distributed environment, be due to a confounding between climate and density (Fig. 3b in the main text). Again, the DD model, which supported my *a priori* expectations, had a higher explanatory power compared to the DI model (Fig. S4.6a), which gave unexpected results. Both the intercept ($\widehat{a_R}$) and the body mass threshold ($\widehat{\gamma_R}$) were positively related to $\hat{\alpha}$, and both were subject to negative density dependence (Table S4.5b,d & Fig. S4.6b,d). The slope for spring body mass ($\widehat{b_R}$) showed negative relationships with environmental skewness, and a positive relationship with density (Table S4.5c & Fig. S4.6c). Judging from the percentage deviance explained all four responses were more affected by density compared to climatic conditions.

Reproductive allocation: reproductive success and offspring body mass
Even though the positive relationship between $\hat{R}$ and $\hat{\alpha}$ was unexpected, $\widehat{rs}$ was negatively related to $\hat{\alpha}$ but positively related to density (Fig. S4.7a & Table S4.6a). Offspring spring and autumn body mass was, however, positively related to $\hat{\alpha}$, but showed evidence of negative density dependence (Fig. S4.7b,c & Table S4.6b,c). As in the normally distributed environments climate seemed to be a more important predictor of these responses than population density (judging from the deviance explained by the DD and DI models, respectively). This means that the relative importance of the DD and DI model was the opposite and in favour of climate for all the 'empirical' measures of reproductive allocation than it was for the 'theoretical' measures presented above.

Somatic allocation: female age body mass
Female age was negatively related to $\hat{\alpha}$, indicating that age was highest when the frequency of good winters was high, whereas female age increased as density increased (Fig. S4.8a & Table S4.7a). In the autumn, female body mass was positively related to $\hat{\alpha}$, and showed clear evidence of negative density dependence (Fig. S4.8b & Table S4.7b). Neither $\hat{\alpha}$ nor density explained spring body mass (Table S4.7c).

Population dynamics: time series analyses
The effect of direct regulation ($\widehat{1-\beta_1}$) showed a curved (concave-down) relationship with $\hat{\alpha}$, being lowest for intermediate values of environmental skewness, and a linear and negative relationship

with density (Fig. S4.9a & Table S4.8a). Delayed regulation ($\widehat{\beta_2}$) was not statistical significantly related to either climate or density (Table S4.8b). The direct effect of climate ($\widehat{\omega_1}$) on population growth was highest, i.e. most negative, at high frequency of poor environments, and the estimated effect of climate in the AR-models increased as density increased (Fig. S4.9c & Table S4.8c). Once again, density was to a high degree explained by climatic conditions (Fig. S4.10).

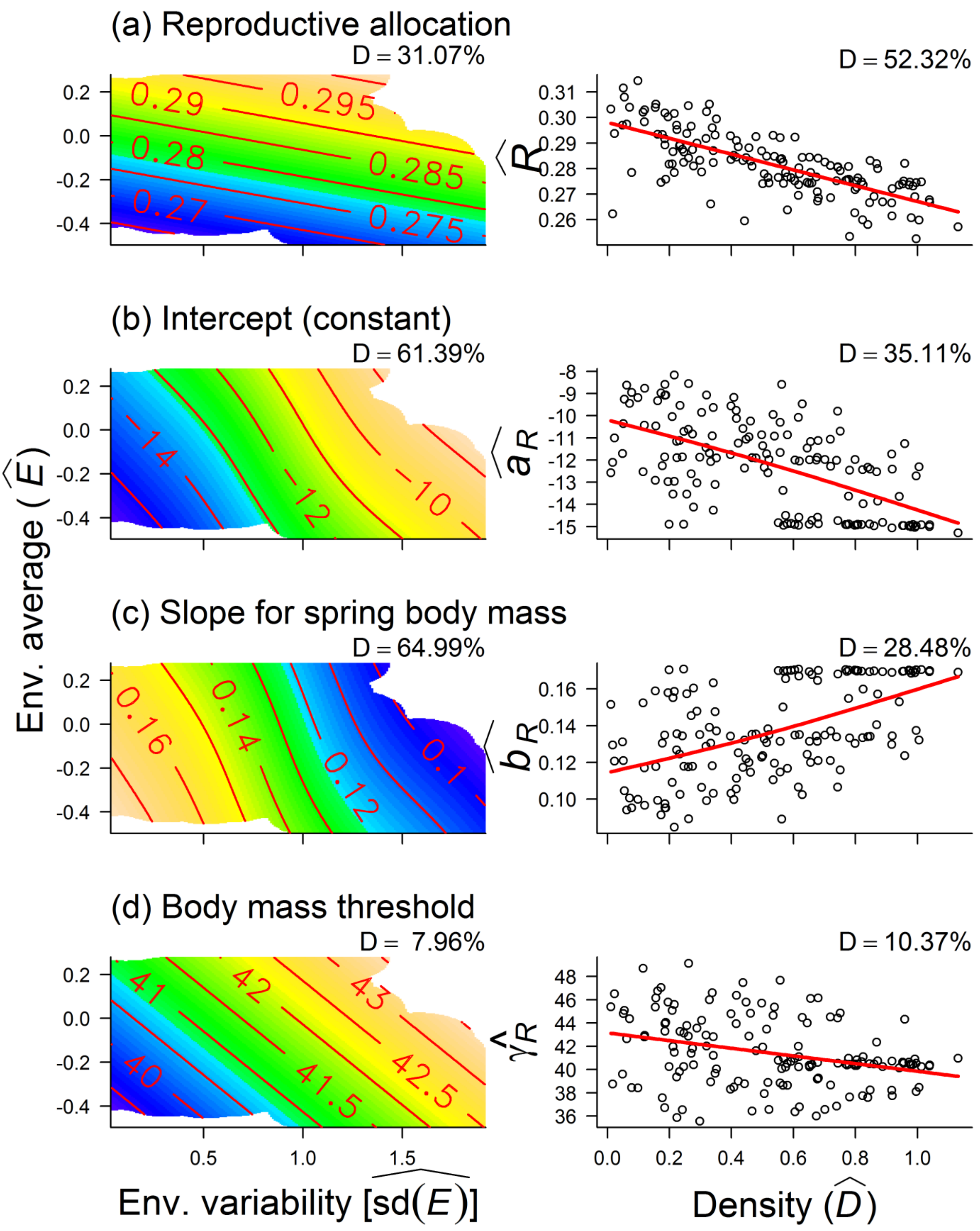

Fig. S4.1. GAMs showing how individual optimization, i.e. (a) how realized reproductive allocation and the (b) intercept, (c) slope and (d) body mass threshold parameters for the reproductive strategy function, was a function of the interaction between environmental variability and average (left panel) and population density (right panel). Table S4.1 provides detailed GAM output.

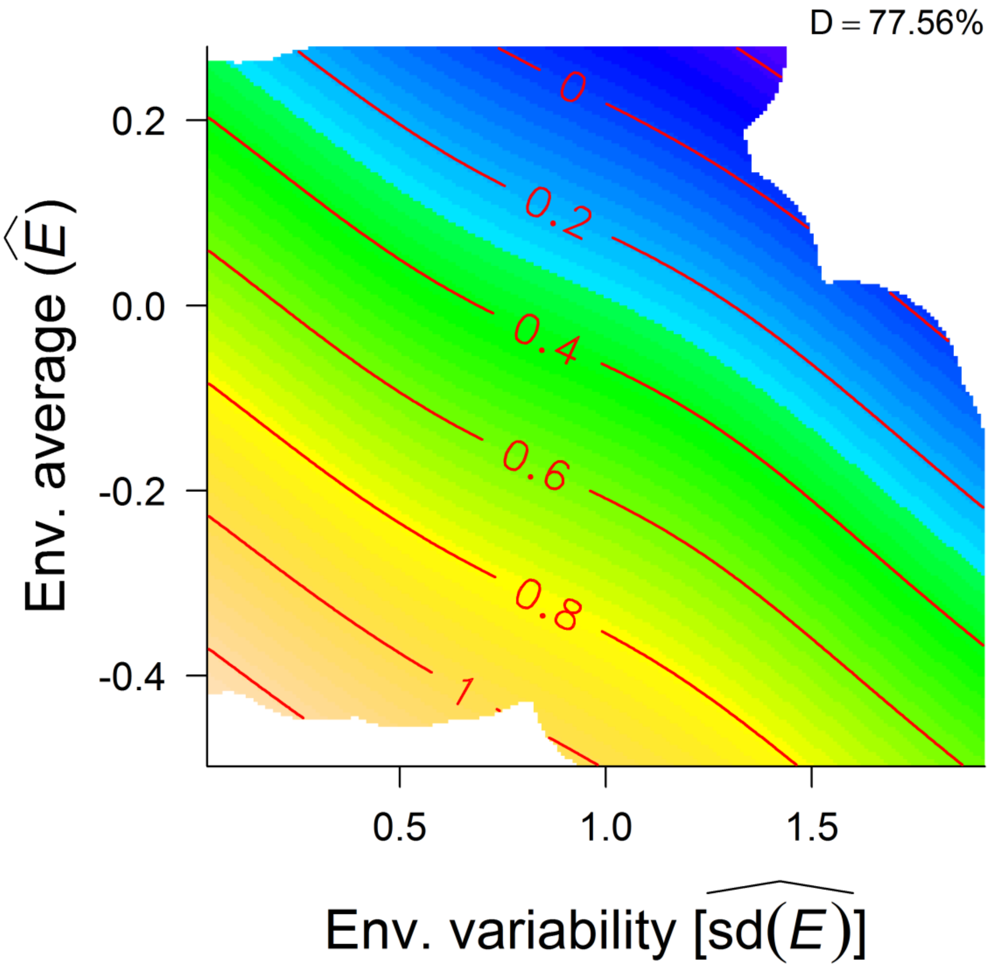

Fig. S4.2. GAMs showing how population density was a function of the interaction between environmental variability and average. Detailed GAM output: Intercept = 0.527 (st. err. = 0.012, *p* <0.001); and estimated degrees of freedom s[$\hat{E}$,$\widehat{\mathrm{sd}(E)}$] = 2.712 (*p* <0.001).

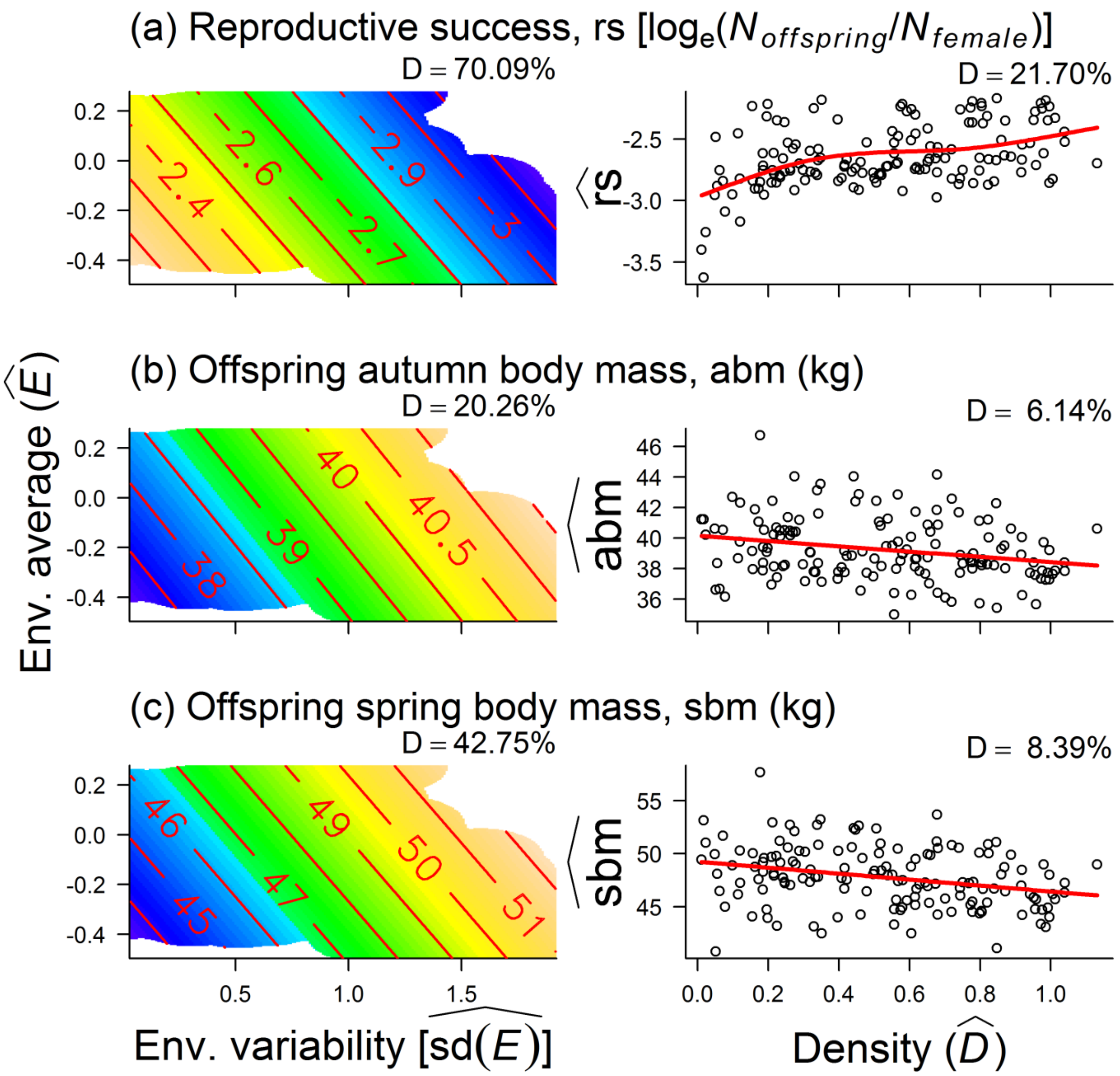

Fig. S4.3. GAMs showing how (a) reproductive success as well as (b) autumn and spring (c) offspring body mass was a function of the interaction between environmental variability and average (left panel) and population density (right panel). Table S4.2 provides detailed GAM output.

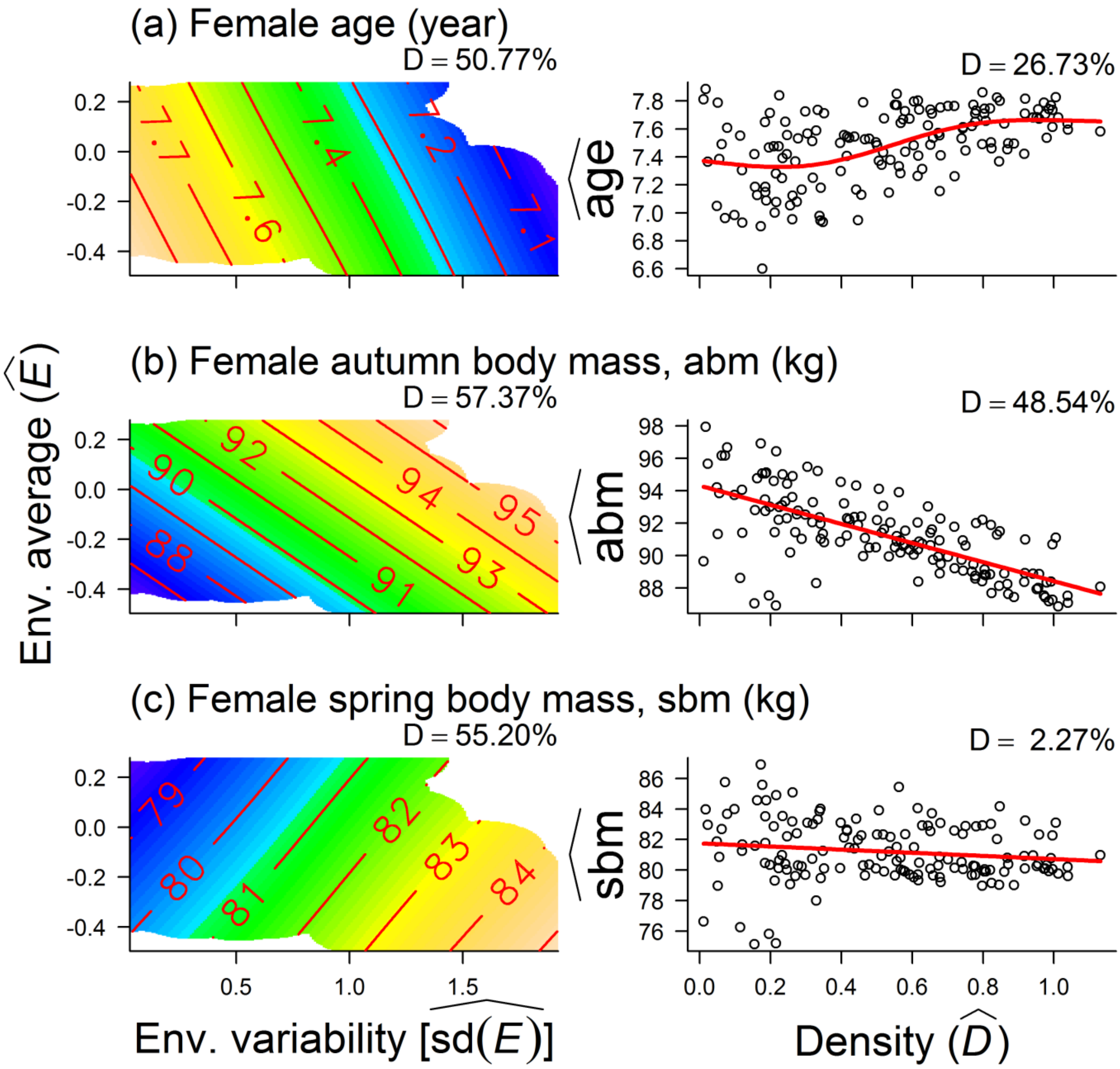

Fig. S4.4. GAMs showing how (a) adult age as well as (b) autumn and spring (c) adult body mass was a function of the interaction between environmental variability and average (left panel) and population density (right panel). Table S4.3 provides detailed GAM output.

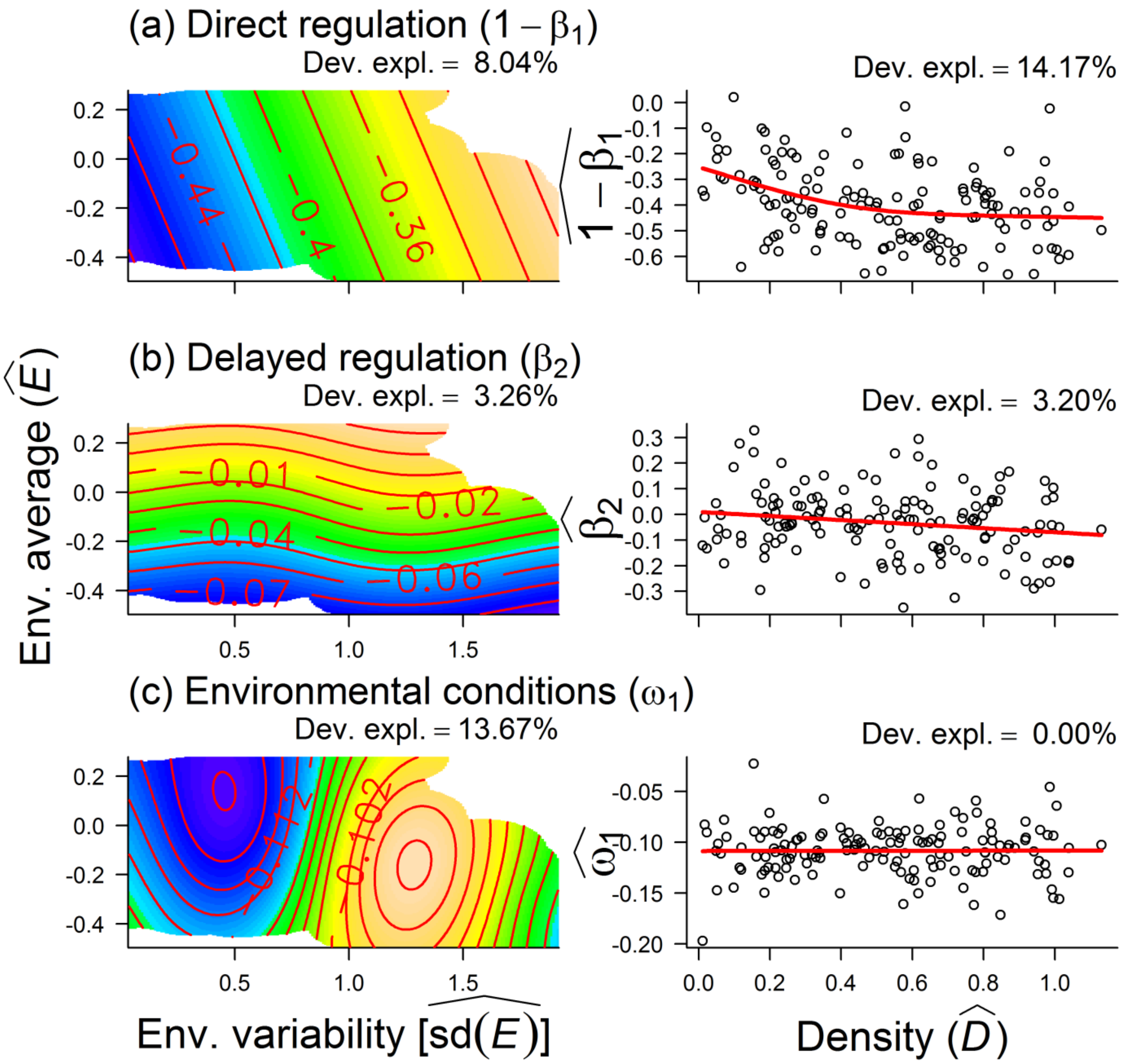

Fig. S4.5. GAMs showing how the estimated coefficient of the ARIMA-model, i.e. (a) direct regulation ($1-\beta_1$), (b) delayed regulation (β_2) and (c) direct effects of climate (ω_1), was a function of the interaction between environmental variability and average (left panel) and population density (right panel). Table S4.5 provides detailed GAM output.

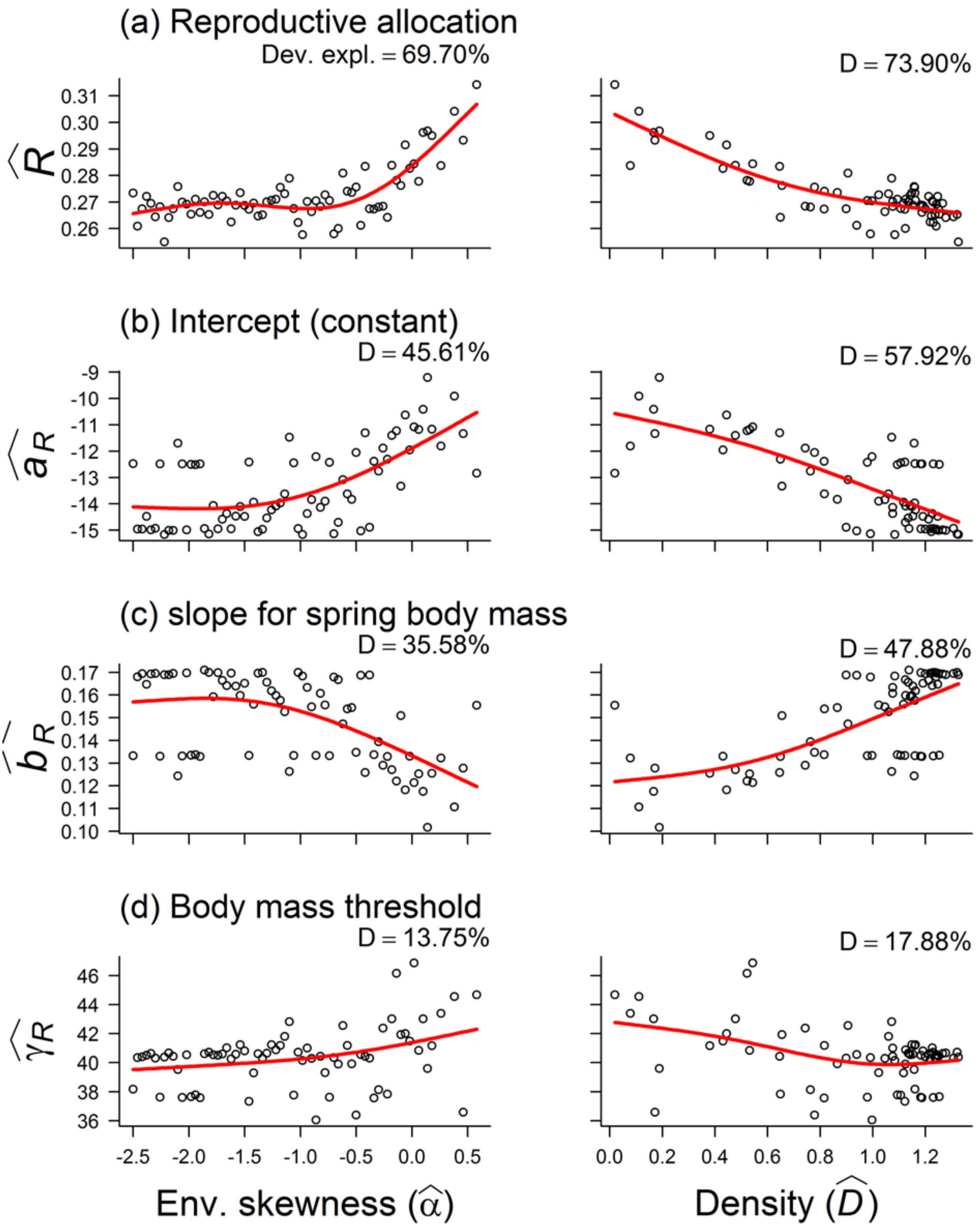

Fig. S4.6. GAMs showing how individual optimization, i.e. (a) how realized reproductive allocation and the (b) intercept, (c) slope and (d) body mass threshold parameters for the reproductive strategy function, was a function of environmental skewness [i.e. the shape parameter ($\hat{\alpha}$) in the skew normal distribution; left panel] and population density (right panel). Table S4.5 provides detailed GAM output. Please note that zero values for $\hat{\alpha}$ represents the standard normal distribution, and represents a baseline for comparison, whereas a negative $\hat{\alpha}$ gives a skew towards the left (negative values for the outcome) and a positive $\hat{\alpha}$ gives a skew towards the right.

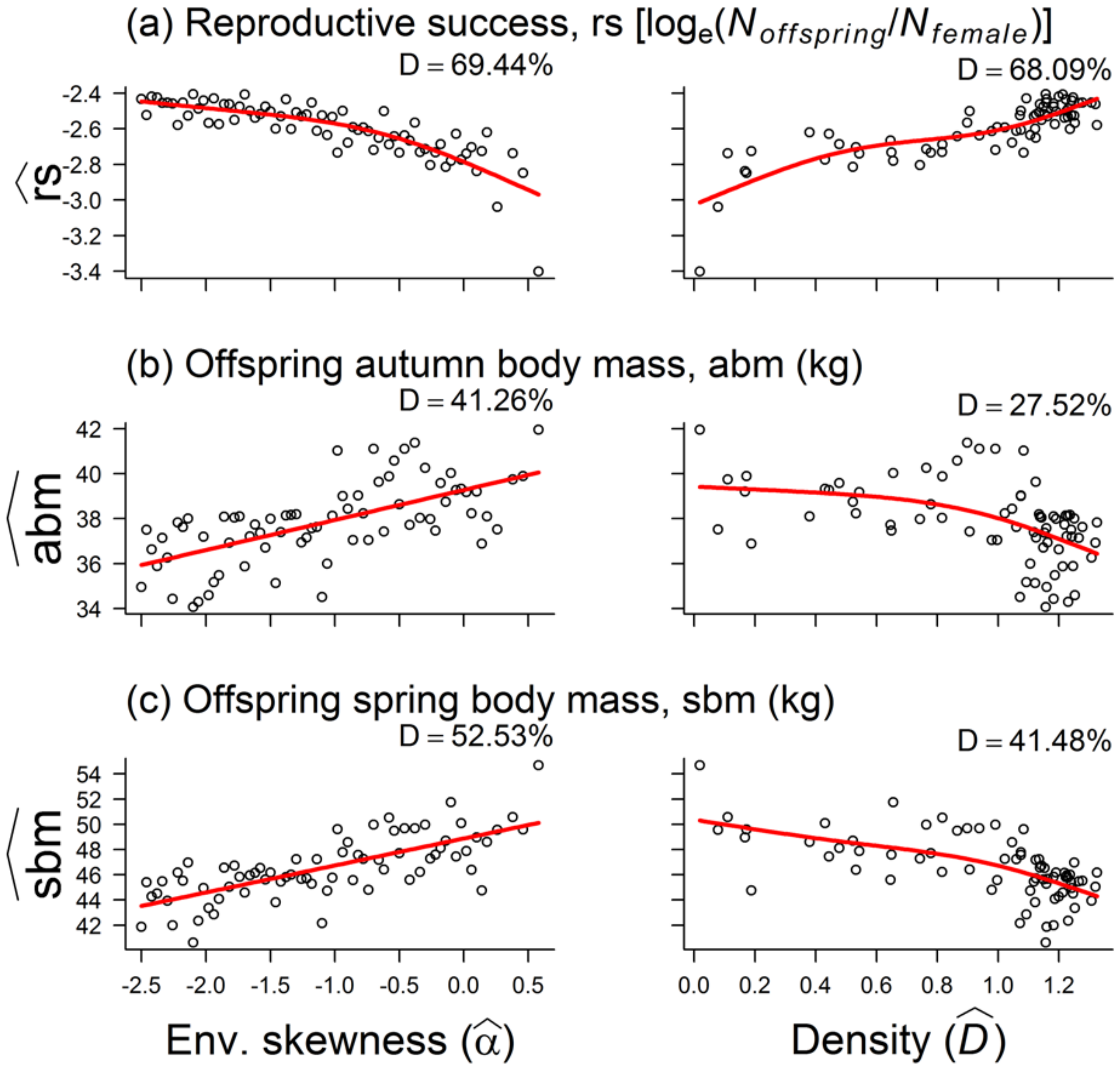

Fig. S4.7. GAMs showing how (a) reproductive success as well as (b) autumn and spring (c) offspring body mass was a function of environmental skewness ($\hat{\alpha}$, see legends in Fig. S4.6 for details; left panel) and population density (right panel). Table S4.7 provides detailed GAM output.

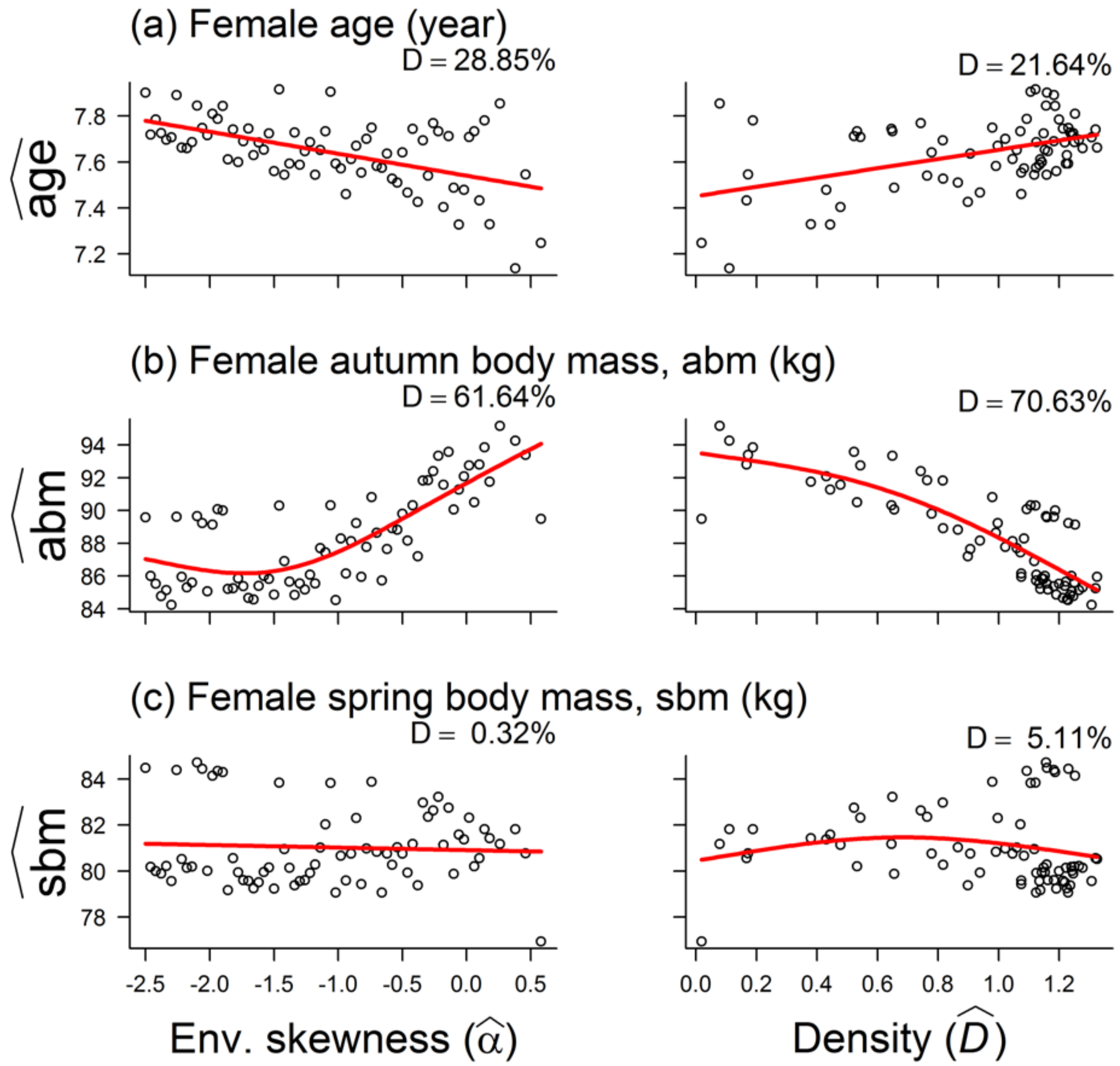

Fig. S4.8. GAMs showing how (a) adult age as well as (b) autumn and spring (c) adult body mass was a function of environmental skewness ($\hat{\alpha}$, see legends in Fig. S4.6 for details; left panel) and population density (right panel). Table S4.8 provides detailed GAM output.

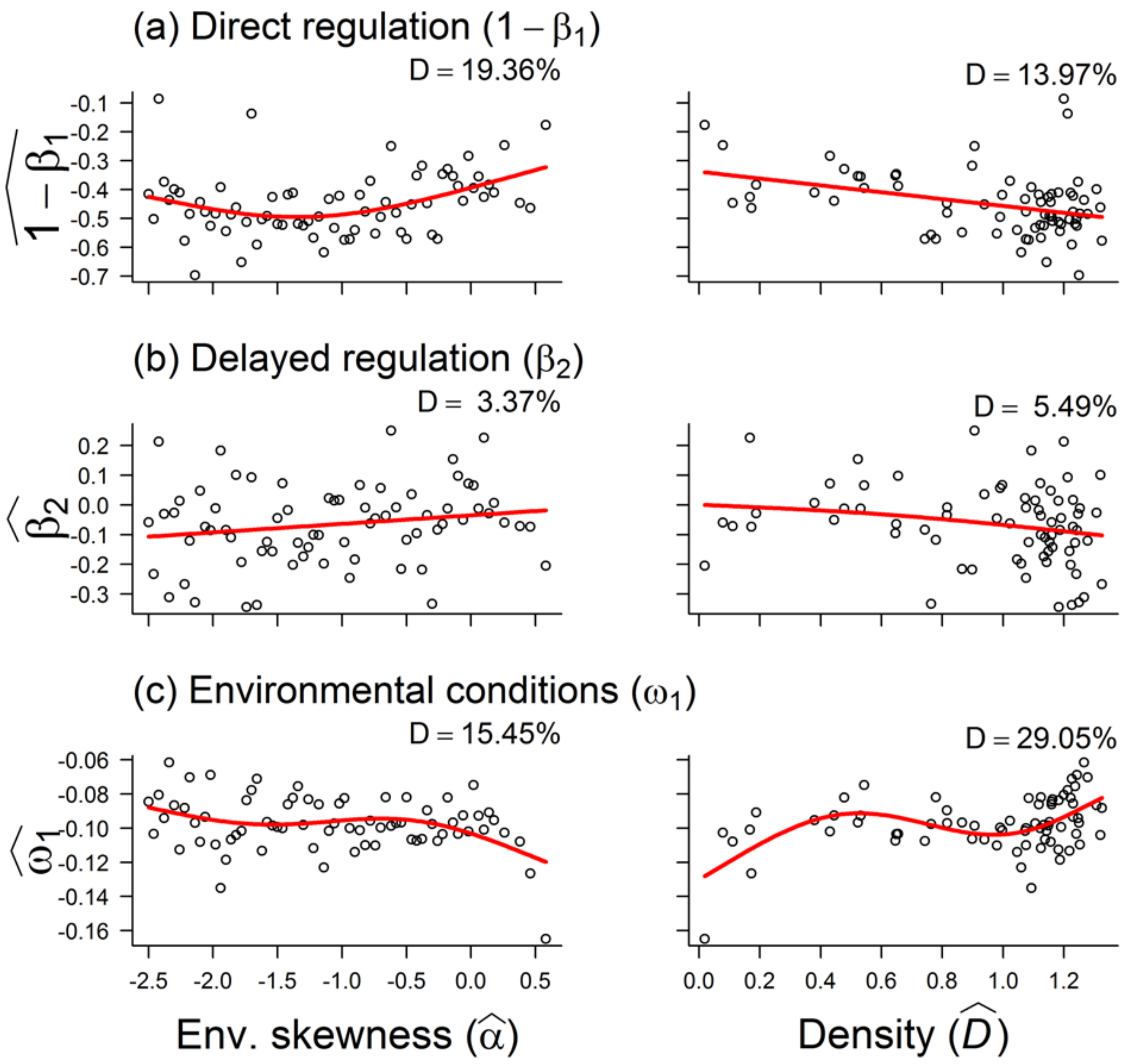

Fig. S4.9. GAMs showing how the estimated coefficient of the ARIMA-model, i.e. (a) direct regulation ($1 - \beta_1$), (b) delayed regulation (β_2) and (c) direct effects of climate (ω_1), was a function of environmental skewness ($\hat{\alpha}$, see legends in Fig. S4.6 for details; left panel) and population density (right panel). Table S4.8 provides detailed GAM output.

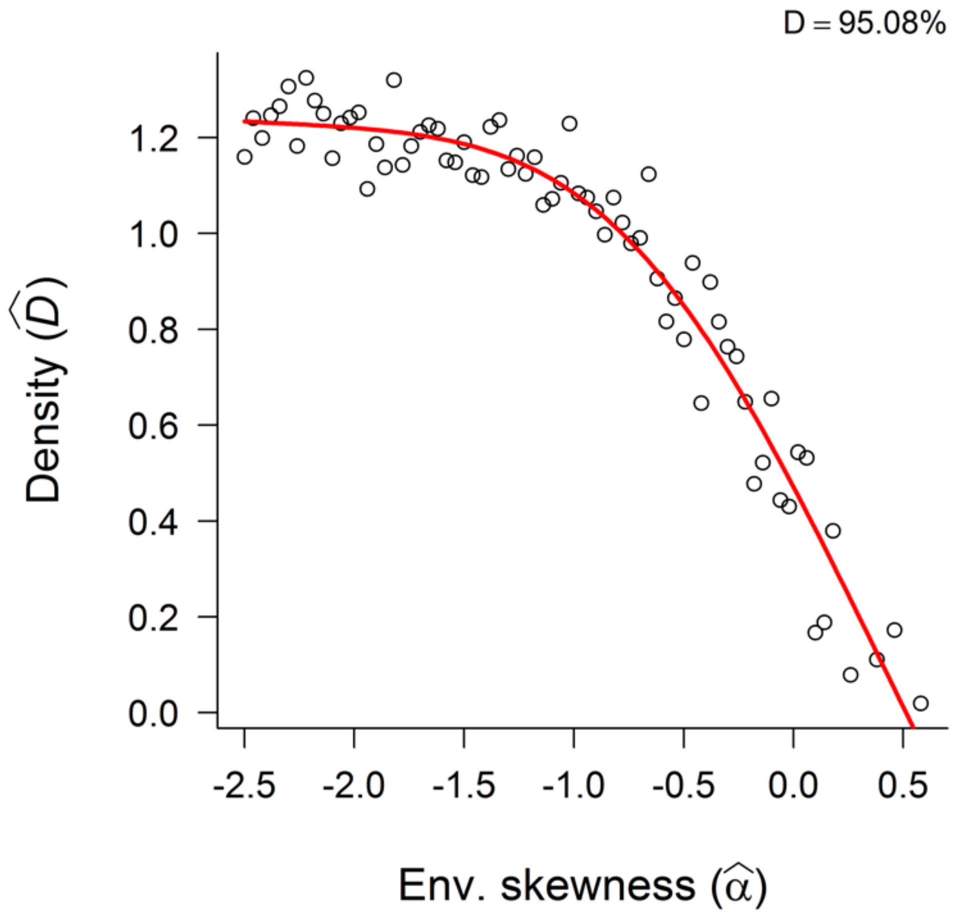

Fig. S4.10. GAM showing how population density was a function of environmental skewness ($\hat{\alpha}$). Detailed GAM output: Intercept = 0.948 (st. err. =0.009, *p* <0.001); and estimated degrees of freedom s($\hat{\alpha}$) = 2.885 (*p* <0.001).

Table S4.1. GAMs showing how individual optimization, i.e. (a) how realized reproductive allocation and the (b) intercept, (c) slope and (d) body mass threshold parameters for the reproductive strategy function, was related to environmental conditions [i; i.e. the interaction between environmental average, $\hat{E}$, and standard deviation, $\widehat{\mathrm{sd}(E)}$] and population density (ii). Estimated degrees of freedom (edf) provide an estimate of the degree of complexity in the relationship, whereas deviance explained (D) provides an estimate of how well the model explains the variance in the response (Fig. S4.1 provides a graphical view of these results).

Parametric coefficients					Smooth terms			
Parameter	Value	St. err	*t*	*p*	Parameter	edf	*F*	*p*
(a) Reproductive allocation, $\hat{R}$								
(i) Winter climate, D = 31.07%								
Intercept	0.282	0.001	327.900	<0.001	s[$\hat{E}$, $\widehat{\mathrm{sd}(E)}$]	2.000	32.460	<0.001
(ii) Density dependence, D = 52.32%								
Intercept	0.282	0.001	395.700	<0.001	s(Density)	1.000	159.100	<0.001
(b) Intercept, $\widehat{a_R}$								
(i) Winter climate, D = 61.39%								
Intercept	-12.242	0.104	-116.700	<0.001	s[$\hat{E}$, $\widehat{\mathrm{sd}(E)}$]	2.695	79.260	<0.001
(ii) Density dependence, D = 35.11%								
Intercept	-12.242	0.135	-90.480	<0.001	s(Density)	1.312	52.710	<0.001
(c) Slope, $\widehat{b_R}$								
(i) Winter climate, D = 64.99%								
Intercept	0.137	0.001	110.100	<0.001	s[$\hat{E}$, $\widehat{\mathrm{sd}(E)}$]	2.747	87.810	<0.001
(ii) Density dependence, D = 28.48%								
Intercept	0.137	0.002	77.360	<0.001	s(Density)	1.366	37.140	<0.001
(d) Body mass threshold, $\widehat{\gamma_R}$								
(i) Winter climate, D = 7.96%								
Intercept	41.414	0.238	173.900	<0.001	s[$\hat{E}$, $\widehat{\mathrm{sd}(E)}$]	2.000	6.229	0.003
(ii) Density dependence, D = 10.37%								
Intercept	41.414	0.234	176.900	<0.001	s(Density)	1.000	16.770	<0.001

Table S4.2. GAM models showing how (a) reproductive success as well as (b) autumn and spring (c) offspring body mass was related to environmental conditions [i; i.e. the interaction between environmental average, $\hat{E}$, and standard deviation, $\widehat{sd(E)}$] and population density (ii). Estimated degrees of freedom (edf) provide an estimate of the degree of complexity in the relationship, whereas deviance explained (D) provides an estimate of how well the model explains the variance in the response (Fig. S4.3 provides a graphical view of these results).

Parametric coefficients					Smooth terms			
Parameter	Value	St. err	*t*	*p*	Parameter	edf	*F*	*p*
(a) Reproductive succes, $\log_e(N_{offspring} N_{female}^{-1})$								
(i) Winter climate, D = 70.09%								
Intercept	-2.643	0.012	-229.900	<0.001	$s[\hat{E}, \widehat{sd(E)}]$	2.000	168.700	<0.001
(ii) Density dependence, D = 21.70%								
Intercept	-2.642	0.019	-141.700	<0.001	s(Density)	2.668	12.850	<0.001
(b) Offspring autumn body mass, kg								
(i) Winter climate, D = 20.26%								
Intercept	39.249	0.151	259.900	<0.001	$s[\hat{E}, \widehat{sd(E)}]$	2.000	18.290	<0.001
(ii) Density dependence, D = 6.14%								
Intercept	39.249	0.163	240.400	<0.001	s(Density)	1.000	9.490	0.002
(c) Offspring spring body mass, kg								
(i) Winter climate, D = 42.75%								
Intercept	47.766	0.178	268.000	<0.001	$s[\hat{E}, \widehat{sd(E)}]$	2.000	53.770	<0.001
(ii) Density dependence, D = 8.39%								
Intercept	47.766	0.225	212.600	<0.001	s(Density)	1.000	13.290	<0.001

Table S4.3. GAM models showing how (a) adult age as well as (b) autumn and spring (c) adult body mass was related to environmental conditions [i; i.e. the interaction between environmental average, $\hat{E}$, and standard deviation, $\widehat{\text{sd}(E)}$] and population density (ii). Estimated degrees of freedom (edf) provide an estimate of the degree of complexity in the relationship, whereas deviance explained (D) provides an estimate of how well the model explains the variance in the response (Fig. S4.4 provides a graphical view of these results).

Parametric coefficients					Smooth terms			
Parameter	Value	St. err	*t*	*p*	Parameter	edf	*F*	*p*
(a) Female age, year								
(i) Winter climate, D = 50.77%								
Intercept	7.486	0.015	489.600	<0.001	s[$\hat{E}, \widehat{\text{sd}(E)}$]	2.149	64.500	<0.001
(ii) Density dependence, D = 26.73%								
Intercept	7.486	0.019	400.400	<0.001	s(Density)	2.780	17.150	<0.001
(b) Female autumn body mass, kg								
(i) Winter climate, D = 57.37%								
Intercept	91.209	0.134	683.200	<0.001	s[$\hat{E}, \widehat{\text{sd}(E)}$]	2.000	96.900	<0.001
(ii) Density dependence, D = 48.54%								
Intercept	91.209	0.146	623.900	<0.001	s(Density)	1.000	136.800	<0.001
(c) Female spring body mass, kg								
(i) Winter climate, D = 55.20%								
Intercept	81.211	0.111	733.300	<0.001	s[$\hat{E}, \widehat{\text{sd}(E)}$]	2.212	72.330	<0.001
(ii) Density dependence, D = 2.27%								
Intercept	81.211	0.163	498.600	<0.001	s(Density)	1.000	3.373	0.068

Table S4.4. GAM models showing how the estimated coefficient of the ARIMA-model, i.e. (a) direct regulation $(1-\beta_1)$, (b) delayed regulation (β_2) and (c) direct effects of climate (ω_1), was related to environmental conditions [i; i.e. the interaction between environmental average, $\hat{E}$, and standard deviation, $\widehat{\mathrm{sd}(E)}$] and population density (ii). Estimated degrees of freedom (edf) provide an estimate of the degree of complexity in the relationship, whereas deviance explained (D) provides an estimate of how well the model explains the variance in the response (Fig. S4.4 provides a graphical view of these results).

Parametric coefficients					Smooth terms			
Parameter	Value	St. err	*t*	*p*	Parameter	edf	*F*	*p*
(a) Direct regulation, 1 - β_1								
(i) Winter climate, D = 8.04%								
Intercept	-0.397	0.012	-33.360	<0.001	s[$\hat{E}, \widehat{\mathrm{sd}(E)}$]	2.000	6.295	0.002
(ii) Density dependence, D = 14.14%								
Intercept	-0.397	0.012	-34.520	<0.001	s(Density)	2.101	8.585	<0.001
(b) Delayed regulation, β_2								
(i) Winter climate, D = 3.26%								
Intercept	-0.032	0.011	-3.035	0.003	s[$\hat{E}, \widehat{\mathrm{sd}(E)}$]	2.315	1.593	0.196
(ii) Density dependence, D = 3.20%								
Intercept	-0.032	0.011	-3.048	0.003	s(Density)	1.000	4.799	0.030
(c) Direct effect of climate, ω_1								
(i) Winter climate, D = 13.67%								
Intercept	-0.108	0.002	-57.410	<0.001	s[$\hat{E}, \widehat{\mathrm{sd}(E)}$]	2.887	7.039	<0.001
(ii) Density dependence, D = 0.00%								
Intercept	-0.108	0.002	-53.700	<0.001	s(Density)	1.000	0.003	0.955

Table S4.5. GAM models showing how individual optimization, i.e. (a) how realized reproductive allocation and the (b) intercept, (c) slope and (d) body mass threshold parameters for the reproductive strategy function, was a function of environmental skewness [i.e. the shape parameter ($\hat{\alpha}$) in the skew normal distribution; left panel] and population density (ii). Estimated degrees of freedom (edf) provide an estimate of the degree of complexity in the relationship, whereas deviance explained (D) provides an estimate of how well the model explains the variance in the response (Fig. S4.6 provides a graphical view of these results).

Parametric coefficients					Smooth terms			
Parameter	Value	St. err	t	p	Parameter	edf	F	p
(a) Reproductive allocation, $\hat{R}$								
(i) Environmental skewness, D = 69.70%								
Intercept	0.273	0.001	382.500	<0.001	s($\hat{\alpha}$)	2.911	52.710	<0.001
(ii) Density dependence, D = 73.90%								
Intercept	0.273	0.001	414.200	<0.001	s(Density)	2.243	73.080	<0.001
(b) Intercept, $\widehat{a_R}$								
(i) Environmental skewness, D = 45.61%								
Intercept	-13.324	0.136	-97.810	<0.001	s($\hat{\alpha}$)	2.177	21.670	<0.001
(ii) Density dependence, D = 57.92%								
Intercept	-13.324	0.120	-111.500	<0.001	s(Density)	1.735	44.650	<0.001
(c) Slope, $\widehat{b_R}$								
(i) Environmental skewness, D = 35.58%								
Intercept	0.149	0.002	81.220	<0.001	s($\hat{\alpha}$)	2.110	14.260	<0.001
(ii) Density dependence, D = 47.88%								
Intercept	0.149	0.002	90.400	<0.001	s(Density)	1.943	26.430	<0.001
(d) Body mass threshold, $\widehat{\gamma_R}$								
(i) Environmental skewness, D = 13.75%								
Intercept	40.407	0.228	177.000	<0.001	s($\hat{\alpha}$)	1.677	5.058	0.008
(ii) Density dependence, D = 17.88%								
Intercept	40.407	0.224	180.500	<0.001	s(Density)	2.297	5.465	0.003

Table S4.6. GAM models showing how (a) reproductive success as well as (b) autumn and spring (c) offspring body mass was a function of environmental skewness [i.e. the shape parameter ($\hat{\alpha}$) in the skew normal distribution; left panel] and population density (ii). Estimated degrees of freedom (edf) provide an estimate of the degree of complexity in the relationship, whereas deviance explained (D) provides an estimate of how well the model explains the variance in the response (Fig. S4.7 provides a graphical view of these results).

Parametric coefficients					Smooth terms			
Parameter	Value	St. err	*t*	*p*	Parameter	edf	*F*	*p*
(a) Reproductive succes, $\log_e(N_{\text{offspring}} N_{\text{female}}^{-1})$								
(i) Environmental skewness, D = 69.44%								
Intercept	-2.603	0.011	-243.900	<0.001	s($\hat{\alpha}$)	2.477	55.510	<0.001
(ii) Density dependence, D = 68.09%								
Intercept	-2.603	0.011	-238.200	<0.001	s(Density)	2.774	48.680	<0.001
(b) Offspring autumn body mass, kg								
(i) Environmental skewness, D = 41.26%								
Intercept	37.846	0.161	235.000	<0.001	s($\hat{\alpha}$)	1.000	49.170	<0.001
(ii) Density dependence, D = 27.52%								
Intercept	37.846	0.180	210.000	<0.001	s(Density)	2.014	10.660	<0.001
(c) Offspring spring body mass, kg								
(i) Environmental skewness, D = 52.53%								
Intercept	46.586	0.205	226.800	<0.001	s($\hat{\alpha}$)	1.000	77.470	<0.001
(ii) Density dependence, D = 41.48%								
Intercept	46.590	0.230	202.500	<0.001	s(Density)	2.170	18.940	<0.001

Table S4.7. GAM models showing how (a) adult age as well as (b) autumn and spring (c) adult body mass was a function of environmental skewness [i.e. the shape parameter ($\hat{\alpha}$) in the skew normal distribution; left panel] and population density (ii). Estimated degrees of freedom (edf) provide an estimate of the degree of complexity in the relationship, whereas deviance explained (D) provides an estimate of how well the model explains the variance in the response (Fig. S4.8 provides a graphical view of these results).

Parametric coefficients					Smooth terms			
Parameter	Value	St. err	*t*	*p*	Parameter	edf	*F*	*p*
(a) Female age, year								
(i) Environmental skewness, D = 28.85%								
Intercept	7.643	0.015	504.600	<0.001	s($\hat{\alpha}$)	1.000	28.380	<0.001
(ii) Density dependence, D = 21.64%								
Intercept	7.643	0.016	480.800	<0.001	s(Density)	1.000	19.330	<0.001
(b) Female autumn body mass, kg								
(i) Environmental skewness, D = 61.64%								
Intercept	88.354	0.221	400.200	<0.001	s($\hat{\alpha}$)	2.594	36.540	<0.001
(ii) Density dependence, D = 70.63%								
Intercept	88.354	0.193	459.100	<0.001	s(Density)	2.082	66.520	<0.001
(c) Female spring body mass, kg								
(i) Environmental skewness, D = 0.32%								
Intercept	81.025	0.199	407.700	<0.001	s($\hat{\alpha}$)	1.000	0.222	0.639
(ii) Density dependence, D = 5.11%								
Intercept	81.025	0.195	415.500	<0.001	s(Density)	1.000	1.187	0.310

Table S4.8. GAM models showing how the estimated coefficient of the ARIMA-model, i.e. (a) direct regulation $(1 - \beta_1)$, (b) delayed regulation (β_2) and (c) direct effects of climate (ω_1), was a function of environmental skewness [i.e. the shape parameter $(\hat{\alpha})$ in the skew normal distribution; left panel] and population density (ii). Estimated degrees of freedom (edf) provide an estimate of the degree of complexity in the relationship, whereas deviance explained (D) provides an estimate of how well the model explains the variance in the response (Fig. S4.9 provides a graphical view of these results).

Parametric coefficients					Smooth terms			
Parameter	Value	St. err	*t*	*p*	Parameter	edf	*F*	*p*
(a) Direct regulation, 1 - β_1								
(i) Environmental skewness, D = 13.36%								
Intercept	-0.450	0.012	-37.770	<0.001	s($\hat{\alpha}$)	2.209	5.983	0.002
(ii) Density dependence, D = 13.97%								
Intercept	-0.450	0.012	-36.880	<0.001	s(Density)	1.000	11.360	0.001
(b) Delayed regulation, β_2								
(i) Environmental skewness, D = 3.37%								
Intercept	-0.066	0.016	-4.231	<0.001	s($\hat{\alpha}$)	1.000	2.443	0.123
(ii) Density dependence, D = 5.49%								
Intercept	-0.066	0.015	-4.271	<0.001	s(Density)	1.241	2.023	0.145
(c) Direct effect of climate, ω_1								
(i) Environmental skewness, D = 15.45%								
Intercept	-0.097	0.002	-55.460	<0.001	s($\hat{\alpha}$)	2.752	4.044	0.011
(ii) Density dependence, D = 29.05%								
Intercept	-0.097	0.002	-60.470	<0.001	s(Density)	2.913	8.770	<0.001

Appendix S5: Modelling Philosophy – Limitations and Assumptions

All models are subject to a set of assumptions and simplifications, and one major simplification in this study is that real organisms have a much wider behavioural repertoire compared to the artificial individuals in the previous model (Bårdsen *et al.*, 2011:252). Compared to the previous model, however, the strategies employed have been developed in order to mimic evolutionary processes more realistically by imposing fewer constraints on the selection of genotypic traits. This, however, came with the cost of added complexity with respect to both assessing convergence and interpretation. Nonetheless, it is important to remember that the strategies still represents simple behavioural rules – All prime-aged individuals were, for example, assumed to: 1) give birth to a single offspring with a constant birth body mass; 2) have an age-independent reproductive allocation (i.e. no senescence); 3) not change their allocation during a given summer; and 4) they have a constant spring body mass threshold deciding whether to allocate in reproduction at all. Additionally, the SKEW simulations expand the way that climatic conditions change in order to assess an increased frequency of both poor and good winter climatic conditions through simulating it as a skew-normal distribution (Appendix S1). This is in line with the predictions from the literature: future global climate change will most likely result in a shift towards more frequent extreme precipitation events (see Appendix S1 and references therein). The approach used for simulating climate change should mimics some of the predictions for future climate change for Fennoscandia. I also assume no harvest or predation – the only source of mortality was starvation and senescence, which is for most wild populations not a valid assumption.

REFERENCES

Bårdsen, B.-J., Henden, J.-A., Fauchald, P., Tveraa, T. & Stien, A. (2011) Plastic reproductive allocation as a buffer against environmental stochasticity - linking life history and population dynamics to climate. Oikos, **20**, 245-257.
